# Impact of Bariatric Surgery on the Expression of Fertility-Related Genes in Obese Women: A Systematic Review of *LEP*, *LEPR*, *MC4R*, *FTO*, and *POMC*

**DOI:** 10.3390/ijms262110333

**Published:** 2025-10-23

**Authors:** Charalampos Voros, Ioakeim Sapantzoglou, Aristotelis-Marios Koulakmanidis, Diamantis Athanasiou, Despoina Mavrogianni, Kyriakos Bananis, Antonia Athanasiou, Aikaterini Athanasiou, Georgios Papadimas, Ioannis Papapanagiotou, Dimitrios Vaitsis, Charalampos Tsimpoukelis, Maria Anastasia Daskalaki, Vasileios Topalis, Marianna Theodora, Nikolaos Thomakos, Fotios Chatzinikolaou, Panagiotis Antsaklis, Dimitrios Loutradis, Evangelos Menenakos, Georgios Daskalakis

**Affiliations:** 1 1st Department of Obstetrics and Gynecology, ‘Alexandra’ General Hospital, National and Kapodistrian University of Athens, 80 Vasilissis Sofias Avenue, 11528 Athens, Greece; kimsap1990@hotmail.com (I.S.); aristoteliskoulak@gmail.com (A.-M.K.); depy.mavrogianni@yahoo.com (D.M.); tsimpoukelischa@gmail.com (C.T.); md181341@students.euc.ac.cy (M.A.D.); martheodr@gmail.com (M.T.); thomakir@hotmail.com (N.T.); panosant@gmail.com (P.A.); gdaskalakis@yahoo.com (G.D.); 2 IVF Athens Reproduction Center V. Athanasiou, 15123 Maroussi, Greece; diamathan16@gmail.com (D.A.); antoathan16@gmail.com (A.A.); diamathan17@gmail.com (A.A.); 3 King’s College Hospitals NHS Foundation Trust, London SE5 9RS, UK; kyriakos.bananis@nhs.net; 4 Athens Medical School, National and Kapodistrian University of Athens, 15772 Athens, Greece; dr.georgepapadimas@gmail.com (G.P.); gpapamd@hotmail.com (I.P.); vaitsisdim@gmail.com (D.V.); vtopalismd@gmail.com (V.T.); loutradi@otenet.gr (D.L.); evmenenakos@hotmail.com (E.M.); 5 Laboratory of Forensic Medicine and Toxicology, School of Medicine, Aristotle University of Thessaloniki, 54124 Thessaloniki, Greece; fotischatzin@auth.gr; 6 Fertility Institute-Assisted Reproduction Unit, Paster 15, 11528 Athens, Greece

**Keywords:** bariatric surgery, obesity, fertility, *LEP*, *LEPR*, *MC4R*, *FTO*, *POMC*, *ARID1A*, *eNOS*, gene expression, weight loss, reproductive health, precision medicine, genetic screening, weight regain

## Abstract

Obesity is a multifaceted disorder influenced by various factors, with heredity being a significant contributor. Bariatric surgery is the most effective long-term intervention for morbid obesity and associated comorbidities, while outcomes vary significantly across individuals. Recent studies indicate that genetic and molecular determinants, particularly alterations in the leptin–melanocortin signalling pathway involving the fat mass and obesity-associated gene (FTO), pro-opiomelanocortin (POMC), melanocortin 4 receptor (MC4R), leptin (LEP), and leptin receptor (LEPR), influence the efficacy of weight loss and metabolic adaptations post-surgery. This narrative review consolidates evidence from peer-reviewed papers available in PubMed and Scopus until July 2025. The emphasis was on novel research and systematic reviews examining genetic polymorphisms, gene–environment interactions, and outcomes following bariatric procedures such as Roux-en-Y gastric bypass (RYGB) and sleeve gastrectomy (SG). Recent research emphasised the integration of genetic screening and precision medicine models into clinical bariatric workflows. Variants in FTO (e.g., rs9939609), MC4R (e.g., rs17782313), *LEPR*, and *POMC* are associated with diminished weight loss post-surgery, an increased likelihood of weight regain, and reduced metabolic enhancement. Patients with bi-allelic mutations in *MC4R*, *POMC*, or *LEPR* exhibited poor long-term outcomes despite receiving effective physical interventions. Furthermore, genes regulating mitochondrial metabolism (such as *PGC1A*), adipokine signalling (such as *ADIPOQ*), and glucose regulation (such as *GLP1R*) have been demonstrated to influence the body’s response to sugar and the extent of weight gain or loss. Two recent systematic reviews elucidate that candidate gene investigations are beneficial; however, larger genome-wide association studies (GWAS) and machine learning techniques are necessary to enhance predictive accuracy. Integrating genetic and molecular screening with bariatric surgery planning possesses significant therapeutic potential. Genotyping can assist in patient selection, procedural decisions, and medication additions, particularly for those with variants that influence appetite regulation or metabolic flexibility. Advancements in precision medicine, including the integration of polygenic risk scores, omics-based profiling, and artificial intelligence, will enhance the customisation of surgical interventions and extend the lifespan of individuals with severe obesity. The epigenetic regulators of energy balance DNA methylation, histone changes, and microRNAs that may affect individual differences in weight-loss patterns after bariatric surgery are also briefly contextualised. We discuss the concept that epigenetic modulation of gene expression, mediated by microRNAs in response to food and exercise, may account for variations in metabolic outcomes post-surgery.

## 1. Introduction

Obesity is a chronic condition that impacts various systems within the body. It is characterised by energy balance issues, elevated body fat levels, and metabolic dysfunction. This occurs due to intricate interactions among genetic, behavioural, and environmental factors [1]. Obesity has become a global epidemic and is a significant contributor to type 2 diabetes, cardiovascular disease, and nonalcoholic fatty liver disease [2]. Bariatric surgery, particularly RYGB, has emerged as the most effective long-term intervention for severe obesity. A multitude of patients have experienced weight loss and resolution of obesity-related health issues. Significant variability exists among individuals regarding weight loss and maintenance post-surgery, indicating that biological factors, particularly genetic predispositions, may influence surgical outcomes [3].

Heterozygous mutations in the MC4R represent the predominant genetic aetiology of monogenic obesity. MC4R encodes a G protein-coupled receptor (GPCR) located in the hypothalamic paraventricular nucleus. It primarily regulates appetite and energy expenditure via the leptin–melanocortin signalling axis [4,5]. Leptin activates proopiomelanocortin (POMC) neurones in the arcuate nucleus, resulting in the release of α-melanocyte-stimulating hormone (α-MSH). This hormone binds to and activates MC4R, resulting in reduced food consumption and increased thermogenesis. Alterations in MC4R, particularly loss-of-function variants, can disrupt receptor trafficking, ligand binding, or downstream signalling via the Gs/cAMP/PKA pathway [6]. This may diminish the efficacy of satiety signals, resulting in overeating, premature obesity, and resistance to weight reduction. These mutations are unequivocally associated with the onset of obesity; however, scientists continue to deliberate on their impact on RYGB outcomes.

Numerous studies have examined the weight loss of individuals with MC4R variants following RYGB, yet the findings have been inconsistent. Aslan et al. (2010) and Valette et al. (2012) discovered that patients with functionally significant heterozygous MC4R mutations experienced a percentage of excess weight loss (%EWL) equivalent to that of matched controls [5,7]. This indicates that the physiological alterations occurring during RYGB, including modifications in gut hormone secretion, bile acid metabolism, and vagal remodelling, may outweigh the hypothalamic impairments induced by MC4R mutations [5,7]. Conversely, some studies indicate that specific variants (such as V95I, I137T, and L250Q) are associated with diminished weight loss post-surgery. This may be due to the receptors malfunctioning or the brain’s diminished sensitivity to gastrointestinal peptides such as PYY and GLP-1, which are recognised for their action via MC4R-expressing neurones in the brainstem and hypothalamus [8,9].

The fat mass and obesity-associated gene (FTO) has been examined as a significant determinant of body weight and postoperative recovery. FTO produces an α-ketoglutarate-dependent dioxygenase that regulates the demethylation of N6-methyladenosine (m6A) in RNA. This influences the expression of genes associated with adipogenesis and mitochondrial function [10]. The rs9939609 A allele is significantly associated with an increased risk of obesity. It has been associated with increased consumption, diminished satiety, and reduced lipolysis [11]. Research indicates that individuals with this variant experience reduced overall weight loss (TBWL), exhibit increased insulin resistance (as assessed by HOMA-IR), and have a higher propensity for weight regain following RYGB [12]. Bandstein et al. (2015) demonstrated that vitamin D levels can influence the impact of FTO polymorphisms on postoperative weight loss [13]. This indicates a gene-nutrient interaction that may alter the functionality of adipocytes and hormonal activity. FTO expression is significantly elevated in the hypothalamus, particularly in nuclei that detect nutrients. It has also been demonstrated to alter AMP-activated protein kinase (AMPK) signalling, a crucial regulator of cellular energy levels [13].

Similarly, other components of the leptin–melanocortin axis, including *LEP*, *LEPR*, *POMC*, *PCSK1*, and *SIM1*, have been associated with both monogenic and polygenic obesity. *LEPR*, responsible for encoding the leptin receptor, activates JAK2/STAT3 and PI3K signalling pathways in hypothalamic neurones, thereby reducing hunger. Alterations in LEPR, such as rs1137101, may disrupt this signalling cascade, diminishing leptin sensitivity and perpetuating hyperphagia [14,15]. PCSK1, responsible for encoding prohormone convertase 1/3, is crucial for the cleavage of proinsulin and POMC into biologically active peptides. Mutations in this gene can disrupt satiety signalling and endocrine function. Longitudinal studies indicate that individuals with alterations in these genes exhibit reduced %EWL, elevated nadir BMI post-surgery, and increased rates of weight recidivism [16]. Campos et al. (2022) discovered that individuals with variants in the leptin–melanocortin pathway experienced a 12.1% reduction in total body weight loss (TBWL) and a 23% increase in weight regain over a 15-year span following RYGB [17].

Although these results indicate a correlation between genotype and postoperative weight loss, the relationship is not consistently evident or predictable. Certain variants recognised to induce functional issues in vitro may not manifest in clinical outcomes that significantly deviate from expectations, such as specific MC4R or POMC variants [18]. Conversely, certain polymorphisms that appear to exert no detrimental effects on receptor function have been associated with significant alterations in metabolic phenotype. This indicates that interactions between genes, interactions between genes and the environment, epigenetic regulation, and compensatory mechanisms in downstream pathways may all be highly significant [19]. In vitro studies indicate that MC4R variants such as R305Q impede cellular motility on surfaces, whereas variants like I137T influence cAMP synthesis. Nonetheless, these molecular anomalies do not consistently result in identical clinical outcomes [20,21]. This illustrates the complexity of central energy regulation and implies that hormonal alterations post-surgery (such as elevated levels of GLP-1, oxyntomodulin, and bile acids) may circumvent or compensate for these genetic issues via alternative neural or endocrine pathways.

Given the increasing emphasis on precision medicine, it is crucial to understand how genetic variations in appetite-regulating pathways influence bariatric outcomes. This is crucial for identifying individuals at risk prior to surgery and developing personalised treatment strategies [22]. Certain authors advocate for routine genotyping to identify patients predisposed to gradual weight loss or rapid weight regain. Nevertheless, the evidence remains ambiguous due to the disparate study designs, follow-up durations, and methods of variant classification. Furthermore, numerous studies lack standardised phenotyping, robust statistical power, or the capacity to integrate functional molecular data.

An enhanced comprehension of the genetic and molecular foundations of obesity has underscored the pivotal function of the leptin–melanocortin pathway in governing appetite, energy expenditure, and sustained body weight equilibrium. This neuroendocrine circuit integrates signals from adipose tissue and the gastrointestinal system with neuronal networks in the hypothalamus to precisely regulate food intake and energy requirements [23]. Disruptions at different points in this pathway—whether through rare monogenic mutations or prevalent single-nucleotide polymorphisms (SNPs)—can result in phenotypes that vary from severe early-onset obesity to more nuanced differences in satiety, thermogenesis, and adipocyte function, potentially affecting the response to bariatric surgery [24].

The MC4R gene encodes the melanocortin-4 receptor, a seven-transmembrane GPCR expressed predominantly in the paraventricular nucleus (PVN) of the hypothalamus and in brainstem autonomic areas [4]. The traditional activation entails α-melanocyte-stimulating hormone (α-MSH), a derivative of the POMC (pro-opiomelanocortin) gene. Upon binding of α-MSH to MC4R, the Gs protein-coupled adenylate cyclase pathway is activated, resulting in an increase in intracellular cAMP levels. This activates PKA, which subsequently phosphorylates downstream targets such as CREB (cAMP response element-binding protein), altering neuronal excitability and the transcription of anorexigenic neuropeptides [25,26].

Mutations in MC4R may impair one or more of the following processes: receptor synthesis, intracellular transport, plasma membrane localisation, ligand interaction, or intracellular signalling. Loss-of-function mutations have been demonstrated to eliminate cAMP generation, diminish surface expression, or enhance receptor internalisation and degradation [27]. People who are heterozygous for these kinds of variations generally have hyperphagia, do not respond well to leptin signalling, and have a dulled reaction to feeling full. The extent of functional impairment differs according to the mutation [28]. For instance, R305Q obstructs membrane trafficking, I137T affects ligand binding and signal transduction, and C326R disrupts disulphide bond formation essential for structural integrity. Notably, despite these molecular modifications, numerous studies indicate that the robust effects of RYGB—such as increased GLP-1 and PYY secretion, modified vagal afferent signalling, and diminished central inflammation—may partially circumvent hypothalamic deficiencies and re-establish energy equilibrium, enabling even mutation carriers to attain significant weight loss [29].

The FTO gene is another significant gene. It encodes a nuclear RNA demethylase that primarily eliminates N6-methyladenosine (m6A) modifications from mRNA. Modifications to the epigenome influence RNA splicing, stability, and translation, subsequently impacting gene networks that regulate appetite, adipocyte proliferation, and mitochondrial development [30]. The FTO gene is prominently expressed in the ARC of the hypothalamus, where it regulates the transcriptional profiles of orexigenic (e.g., NPY/AgRP) and anorexigenic (e.g., POMC) neurones. The widely studied SNP rs9939609 (A allele) is associated with increased energy intake, preference for high-fat foods, and reduced lipolytic activity in adipocytes, potentially via downregulation of AMPK and upregulation of mTORC1 signalling [31,32]. Environmental and hormonal factors, such as vitamin D levels, appear to influence the functionality of FTO variations. Bandstein et al. showed that people with the AA genotype who were low in vitamin D lost up to 14% less excess BMI after RYGB. This shows how genes and the environment can affect metabolic plasticity [13].

*LEP* and *LEPR* (leptin and its receptor) constitute a fundamental component of the central energy regulatory axis. Leptin, secreted in relation to adipose tissue, traverses the blood–brain barrier and attaches to its receptor (*LEPR*) on hypothalamic neurones, thereby activating the JAK2/STAT3, PI3K/AKT, and ERK1/2 signalling pathways. These cascades suppress orexigenic neurones (e.g., AgRP/NPY) and promote POMC expression, thereby augmenting MC4R-mediated anorexigenic signalling [33,34]. Changes in the *LEPR* gene, such rs1137101 Gln223Arg, can make it harder for the receptor to bind to other molecules or convey signals, which can cause central leptin resistance and long-term overeating. Clinically, *LEPR* dysfunction may manifest as poor responsiveness to diet and pharmacologic therapies, and potentially to bariatric surgery, depending on the extent of central leptin insensitivity [35].

POMC, as the precursor to α-MSH, ACTH, and β-endorphins, requires cleavage by PCSK1 (prohormone convertase 1/3) to yield its biologically active fragments. Changes in either gene could lead to less α-MSH being made, which would then lead to less MC4R being activated [36]. This reduction obstructs the satiety signal originating from the ARC, permitting the urge to eat to persist despite elevated leptin and insulin levels. Such defects may be exacerbated by downstream polymorphisms or transcriptional regulators such as SIM1 (Single-minded homolog 1), which influences MC4R expression and PVN development, or SH2B1, which modulates insulin and leptin receptor signalling [37,38]. Loss-of-function mutations in SH2B1 have been associated with severe early-onset obesity, neurobehavioral disorders, and insulin resistance, emphasising its critical role in coordinating neuroendocrine and metabolic balance [39].

The leptin–melanocortin axis is tightly connected to peripheral metabolic activities, such as insulin secretion (through PI3K/AKT), hepatic glucose output (through AMPK), and adipocyte lipolysis (through PKA and ATGL). Following RYGB, the substantial shift in gut-derived hormonal signals—which increases particularly in GLP-1, PYY, and bile acids—can stimulate these central and peripheral pathways, increasing satiety, glycemic control, and calorie expenditure [40]. Yet, in individuals with specific genetic variants, such as loss-of-function MC4R mutations or FTO risk alleles, the extent of metabolic adaptation may be attenuated, leading to slower or incomplete weight loss, plateauing, or early weight regain. Furthermore, genotype-specific variations in inflammatory signalling, vagal afferent feedback, and hedonic eating behaviour may influence both short-term and long-term results [4,41].

The functional integrity of genes in the leptin–melanocortin pathway—*MC4R*, *FTO*, *LEPR*, *POMC*, *PCSK1*, *SIM1*, and *SH2B1*—is essential for the proper neuroendocrine response after bariatric surgery. These genes control critical pathways such as JAK/STAT, AMPK-mTOR, cAMP-PKA, and ERK1/2, which govern not just hunger and satiety but also adipocyte development, insulin sensitivity, and mitochondrial function [17,22]. Understanding the complicated interplay between these pathways and how they are altered by surgery provides a platform for precision bariatric therapy. Future therapeutics may include preoperative genotyping, customised nutrition plans, or supplementary medicine according to genetic risk profiles.

This systematic review aims to consolidate existing evidence regarding the influence of MC4R, FTO, and associated gene variants in the leptin–melanocortin signalling pathway on weight loss following RYGB. We aim to determine whether specific genetic profiles can forecast individual responses to surgery by integrating clinical data with molecular insights. We aim to elucidate the molecular mechanisms that may account for this variability. This study seeks to develop genotype-guided algorithms for the management of bariatric patients and to enhance our understanding of the neuroendocrine factors that contribute to successful weight loss. In addition to genetic variations, epigenetic mechanisms such as DNA methylation, histone remodelling, and microRNA regulation may influence transcriptional activity within the leptin–melanocortin pathway. These pathways may explain the varying metabolic responses that occur post-surgery. Epigenetic control facilitates metabolic adaptability among individuals in conjunction with genetic heterogeneity. The modulation of gene expression by miRNA is dynamically influenced by food composition and physical activity, therefore creating a mechanistic link between environmental factors and pathway outcomes within the leptin–melanocortin axis.

## 2. Material and Methods

This systematic review adhered to the 2020 guidelines of the Preferred Reporting Items for Systematic Reviews and Meta-Analyses (PRISMA). The protocol was preemptively filed in the PROSPERO database (International Prospective Register of Systematic Reviews) under the registration number (CRD420251074230).

### 2.1. Choosing Studies

The PRISMA 2020 statement guided the study selection process, which is shown in the PRISMA flow diagram (see Section 3.1). The purpose was to find all original research studies that looked at the link between genetic variations, especially those in the leptin–melanocortin pathway, and weight loss after RYGB. Through examining databases and other sources, we found a total of 407 records. Using structured queries that combined MeSH terms and phrases linked to bariatric surgery, obesity-associated genes, and genetic variations, 352 references were found in electronic databases like PubMed, Scopus, and Web of Science. Through citation searching and manually going through reference lists of relevant publications and reviews, we found 55 more entries. After removing duplicates, 75 records were taken out, and another 97 records were left out because they did not match basic eligibility requirements (for example, they were animal studies, abstracts without full text, or themes that were not relevant). This left 180 records that needed to be checked for titles and abstracts. After screening, 125 records were thrown out since they were not relevant. 55 full-text articles were then retrieved and checked to see if they were eligible.

We did a full-text screening and left out 40 articles for the following reasons. There were 24 opinion articles or conference papers that did not have any data that could be extracted. There were 14 reviews or meta-analyses that did not have any original outcomes. There were 12 that did not disclose outcomes by genotype or did not include RYGB patients. In the end, 15 studies met all of the requirements for inclusion and were included in the systematic review. But these 15 studies were actually five different reports, and some of those publications included more than one dataset, follow-up time point, or research group. This difference between the number of studies and reports is because several publications have substudies or analysis of different polymorphisms all in one article or research output.

We utilised Boolean logic and controlled language, including MeSH keywords and free-text, to identify literature examining genetic variants associated with weight reduction during RYGB. The methodology was adapted to conform to the syntax of each database, and filters were employed to yield results just for research on individuals documented in English.

We employed a systematic search methodology across PubMed, Scopus, and Web of Science to ensure that our literature review was comprehensive and replicable. We employed Boolean operators and gene-specific keywords (including *FTO*, *MC4R*, *LEPR*, *SH2B1*, *POMC*, *PCSK1*, and *SIM1*) in conjunction with phrases associated with outcomes such as “weight loss,” “%EWL,” “%TBWL,” and “weight regain.” We applied filters to restrict the results to English-language studies involving humans. Table 1 presents a summary of the specific questions and contexts employed for each database.

The PRISMA diagram shows this difference by listing “Studies included in review: n = 15” and “Reports of included studies: n = 5” separately, as recommended by PRISMA 2020. These five reports gave us separate, analysable data sets from a variety of groups and methods. The registered protocol in the PROSPERO database (Registration ID: CRD420251074230) supervised this systematic and strict selection procedure. This made sure that the review was open and could be repeated.

### 2.2. Eligibility Criteria

This systematic review was conducted to examine the association between germline genetic variants and weight loss results following Roux-en-Y gastric bypass, a highly effective and hormonally active kind of bariatric surgery. Eligible studies examined individuals with obesity (BMI ≥ 35 with comorbidities or ≥40) who underwent Roux-en-Y gastric bypass (RYGB). Researchers examined papers that encompassed alternative bariatric surgeries, such as VSG, BPD, or AGB, solely when the outcomes for RYGB significantly diverged from those of the other procedures. This is significant as RYGB alters GLP-1, PYY, GIP, BA circulation, gut microbiota composition, and CNS feedback mechanisms, which are recognised to influence genotype-dependent metabolic pathways.

Genotyping for variants, including SNPs or rare loss-of-function mutations, must be conducted in genes directly involved in the LEP–MC regulatory network. The components included MC4R (a receptor regulating appetite suppression and energy expenditure), *FTO* (a demethylase influencing food intake), *LEPR* (a receptor for leptin that modulates leptin sensitivity and signalling to the arcuate nucleus), *POMC* (a precursor for ACTH and α-MSH that activates MC4R), PCSK1 (an enzyme that converts POMC into active peptides), SH2B1 (an intracellular signal transducer within the leptin-insulin-insulin receptor axis), and SIM1 (a transcription factor in the hypothalamus essential for paraventricular nucleus function and subsequent MC4R expression). These genes are crucial for the central nervous system to integrate food information and hormonal responses to blood sugar fluctuations.

We exclusively sought trials that documented anthropometric or metabolic outcomes following RYGB, categorised by genotype. %TBWL, %EWL, ΔBMI, or WR were required to be included in these outcomes. We also examined papers that identified gene-specific variations in T2DM remission, HTN management, NAFLD resolution, or IR reversal, provided these outcomes were explicitly associated with the surgical–genetic interaction. Incorporating trials with a minimum mid-term follow-up (≥6 months) ensured accurate interpretation of weight loss trajectories and metabolic sustainability.

We excluded articles that did not do genetic stratification or direct DNA variant analysis. This systematic review excluded papers that just examined transcriptomics, DNA methylation, miRNA expression, or other non-genomic regulatory variables. We excluded papers that did not differentiate RYGB outcomes from other bariatric surgery types or that aggregated patients without comparing their genotypes. This was implemented to prevent confusion arising from variations in surgical procedures or other non-genetic influences.

We excluded juvenile cohorts, individuals with syndromic obesity (such as Prader–Willi syndrome or Bardet–Biedl syndrome), and instances of secondary obesity (such as those resulting from Cushing’s illness or central nervous system tumours) due to their distinct pathophysiology and gene–phenotype associations. Reviews, editorials, non-peer-reviewed articles, and conference abstracts were excluded due to the absence of extractable source data.

The included research employed rigorous yet physiologically valid criteria to elucidate the impact of specific alterations in the LEP–MC–INS–BA signalling cascade on the magnitude and durability of weight loss following RYGB. This precision medicine approach enabled the identification of surgical responders and non-responders with distinct genotypes. This may facilitate PGx-based selection for BS in the future.

### 2.3. Sources of Information and Search Methodologies

We conducted a comprehensive literature search employing a structured, tiered approach to identify all publications examining the relationship between genetic variants and weight loss outcomes following Roux-en-Y gastric bypass. We examined three major biological databases PubMed/MEDLINE, Scopus (Elsevier), and WoS (Clarivate Analytics) from their inception until April 2025. We selected these databases because to their extensive information on clinical, molecular, and genomic studies pertinent to obstetrics, biosciences, and pharmacogenomics.

The LS was designed to identify papers linking SNPs or atypical LoF changes in genes along the LEP–MC–INS–CNS axis to the efficacy of WL following RYGB. No filters were available for date, country, or study design. Only full-text, peer-reviewed publications published in English are eligible for inclusion. This ensured that the approaches were explicit and reproducible.

The search methodology was predicated on three fundamental concepts: (1) surgical intervention (exclusively RYGB), (2) genetic or molecular determinants (such as *MC4R*, *FTO*, *LEPR*, *SH2B1*, *PCSK1*), and (3) postoperative outcomes (including %TBWL, %EWL, ΔBMI, WR, T2DM remission, and IR reversal).

This is an illustration of a PubMed search query: (“RYGB” OR “gastric bypass”) AND (“MC4R” OR “FTO” OR “LEPR” OR “POMC” OR “PCSK1” OR “SH2B1” OR “SIM1”) AND (“SNP” OR “variant” OR “mutation”) AND (“%TBWL” OR “%EWL” OR “BMI change” OR “WR” OR “T2DM”).

We modified the search terms Socrates, Emtree, and WoS to align with the indexing systems of each database. This ensured that both genomic and clinical terminology were highly sensitive. All identified records were imported into EndNote to eliminate duplication. Subsequently, Rayyan (QCRI) was employed to blind-screen the documents, facilitating the examination of titles, abstracts, and full contents.

To enhance comprehensiveness, we conducted manual backward citation tracking (utilising the references of included papers) and forward citation searching (employing Google Scholar and Scopus). This systematic approach facilitated the discovery of articles that may have been overlooked, including older studies and preliminary molecular investigations that may not be adequately indexed.

The screening technique for each discovered record consisted of two steps. The title and abstract were initially evaluated for relevance. The entire text was examined to determine compliance with the criteria outlined in Section 3.2. Two reviewers with expertise in biological sciences and medical genetics independently conducted all screening procedures. Disagreements were resolved through consensus discussions or by a third reviewer with PGx expertise rendering a conclusion.

Eligible studies were required to present RYGB-specific outcomes categorised by genetic profile. Particularly, research examining SNPs or rare variants in genes regulating signalling between the CNS, stomach, and adipose tissue were prioritised. The selected loci (*MC4R*, *FTO*, *LEPR*, *POMC*, *PCSK1*, *SH2B1*, *SIM1*) were identified due to their significance in neurohormonal regulation of hunger/satiety, energy expenditure, fat distribution, and insulin sensitivity. The hormonal alterations induced by RYGB significantly influence these processes (e.g., GLP-1, PYY, GIP, BA).

The objective of this SR was to compile a dataset of high-quality research linking genotype to weight loss variability after RYGB by integrating a molecularly informed LS methodology with rigorous screening techniques. This evidence base provides critical insights for the future PGx-guided classification of surgical candidates and elucidates the variations in metabolic alterations post-bariatric surgery among individuals.

### 2.4. The Data Acquisition Process

A systematic and standardised DE framework was employed to ensure the repeatability, accuracy, and comprehensiveness of this SR. Two reviewers, both experienced in BS and PGx, concurrently conducted DE utilising a special form designed for gene–WL investigations post-BS. We utilised validated templates from prior BS–SNP assessments to create this form, and we conducted a pilot test to ensure clarity and consistency. During DE, all discrepancies were resolved by dialogue. In the event of ongoing disagreement, a third reviewer possessing expertise in CNS–LEP–MC signalling rendered the ultimate conclusion.

We documented the bibliographic details (first author, publication year, research location, and journal) for each qualifying paper, together with the study strategy (prospective, retrospective, case-control, or hybrid) and the sample size (N). We obtained data regarding the population, including the average baseline BMI (kg/m^2^), age (years), sex distribution (M:F ratio or %F), and follow-up duration (months/years). Explicit I/E criteria were observed to assess the homogeneity of the samples. We included mixed-BS cohorts just if they provided distinct results for RYGB. We exclusively incorporated studies that concentrated on RYGB.

The primary emphasis of genetic data extraction was on significant CNS–OB–WL genes such as *MC4R*, *FTO*, *LEPR*, *SH2B1*, *PCSK1*, *POMC*, and *SIM1.* The specific SNP for each gene was recorded using its rsID (e.g., rs9939609, rs17782313, rs1137101), along with the corresponding genotypes (e.g., AA vs. AT vs. TT), MAF (if applicable), and genetic model (dominant, recessive, additive). To verify the accuracy of the data, genotyping methods such as TaqMan qPCR, SNP arrays (e.g., Illumina), or whole genome/exome sequencing (WGS/WES) were documented. Studies demonstrating Hardy–Weinberg equilibrium compliance, genotyping call rates exceeding 95%, and stringent quality control requirements were considered to possess superior quality.

We eliminated surgical variables to ensure uniformity throughout all surgeries. Only patients who underwent RYGB were included; those who had VSG, BPD, or AGB were excluded unless the findings for RYGB were distinctly delineated. We documented numerous details regarding the surgery, including whether it was laparoscopic or open access, the length of the limbs, and the type of metabolic care required by the patient postoperatively.

The primary outcomes of interest were %EWL, %TBWL, ΔBMI (from baseline to post-RYGB), and WR (% increase from nadir). Alterations in metabolic comorbidities post-surgery, including ΔT2DM status, ΔIR, and ΔHTN, were classified as secondary outcomes. We exclusively examined studies that presented results categorised by genotype or allele carrier status. We recorded the evaluation intervals (e.g., 6 months, 12 months, 24 months) to facilitate temporal comparison.

We obtained the effect sizes (β coefficients, odds ratios, hazard ratios), the corresponding 95% confidence intervals, and the p-values for genotype–phenotype associations. We examined studies to see if they employed multivariate models that considered potential confounders such as age, sex, baseline BMI, diabetes mellitus status, or follow-up duration. When such modifications were not implemented, they were identified as a potential source of bias.

All data were consolidated into a central database that performed self-verification twice. In instances of issues or absent genotype-level results, we attempted to contact the authors via email. We excluded studies that lacked extractable genotype-stratified outcomes or failed to demonstrate a correlation between SNP and RYGB outcomes.

This comprehensive DE method ensured accurate documentation of genotype-WL-RYGB associations, emphasising genes within the *CNS-LEP-MC-INS-BA* axis. The database generated from this work served as the foundation for assembling and critically evaluating the varied responses of individuals to RYGB from a pharmacogenomic standpoint.

We used the NOS to check the methodological quality of the observational studies we included. This is a common way to check for bias in non-randomised research. This tool looks at three areas: choosing study groups (4 points), making sure the groups are comparable (2 points), and figuring out the outcome (3 points). Two reviewers looked at each study on its own. There were differences that were settled by agreement or by talking to a third reviewer. Table 2 shows the scores for each study and each domain in detail. We put studies into three groups based on their scores: low risk of bias (7 or higher), moderate risk (5–6), and high risk (below 5).

### 2.5. Assessment of Bias and Quality Control

To meticulously evaluate the methodological rigour and internal validity of the included papers, we employed the NOS, a structured instrument designed for non-randomised studies such as retrospective cohorts, prospective observational designs, and case–control analyses frequently utilised in biospecimen pharmacogenomics research. Two reviewers, independently trained in SR method, PGx, and the outcomes of metabolic surgery, employed the NOS to evaluate each report that satisfied the criteria. The NOS examines research in three critical domains: SEL, COMP, and OUT. Each domain examines a distinct form of bias. R3, a seasoned PGx specialist, facilitated the resolution of disputes among reviewers via dialogue or third-party arbitration.

In the SEL domain, evaluations were based on the clarity and precision with which the BS population was delineated. To achieve maximum points, research must demonstrate RYGB efficacy (rather than aggregating bariatric surgery types), present a non-syndromic obesity cohort, and incorporate baseline anthropometric and metabolic data such as BMI (kg/m^2^), T2DM prevalence, age, and sex ratio. Studies that sequentially recruited patients, conducted SNP profiling before to surgery, and initiated follow-up concurrently post-surgery were favoured. The amalgamation of RYGB outcomes with VSG, AGB, or BPD, or the inclusion of specific genotypes such as MC4R-mut carriers, rendered the results less generalisable.

In the COMP domain, the SNP–outcome associations were meticulously examined to ensure their validity. This involved assessing whether the studies appropriately conducted the multivariable analysis to account for factors such as age, sex, baseline BMI, type 2 diabetes mellitus or insulin resistance status, and follow-up duration. Research employing generalised linear models, logistic regression, or Cox proportional hazards models that considered these characteristics achieved superior ratings. Research employing gene × environment (G × E) interaction terms, genotype-stratified analysis (e.g., AA versus AT/TT), or model-specific coding (dominant, recessive, or additive) received greater emphasis. Inclusion of 95% confidence intervals and *p*-values alongside effect estimates was essential. Studies that conducted superficial comparisons without modifications (for instance, comparing ΔBMI averages among genotypes without considering sex or baseline BMI) had a reduced COMP score.

The OUT domain examined the clarity, consistency, and objectivity of the assessment of postoperative weight loss and metabolic outcomes. Robust research explicitly delineated %EWL (for instance, %EWL = [(initial weight − current weight)/(initial weight − target weight)] × 100), %TBWL, ΔBMI, or WR utilising established cutoffs (for example, ≥25% weight regain from nadir weight). A minimum follow-up duration of six months was required to ensure the stability of the results. Follow-up durations of 12 months or longer were favoured, with reports issued biannually (for instance, 6 m + 12 m + 24 m). Research utilising clinic-verified weights or electronic health record data achieved superior scores compared to studies dependent on self-reported results. The trials were examined for outcome data categorised by genotype. If a study addressed SNP analysis without presenting stratified WL values (such as %TBWL by FTO-rs9939609 genotype), the OUT domain score was diminished. OUT also declined due to inadequate follow-up or selective attrition, like as dropouts from a certain genotype group.

Each study received a NOS score, which varied from 0 to 9. The classification thresholds were L-RoB (≥7), M-RoB (5–6), and H-RoB (≤4). We documented the individual domain ratings (SEL/COMP/OUT) to identify trends of methodological robustness or deficiencies throughout the included research. We constructed a table of the NOS results for explicit reporting (refer to Table 3) and employed summary trends to enhance the narrative synthesis’s significance. SNP–RYGB associations from H-RoB studies, such as LEPR-rs1137101 and WR, were meticulously examined and not extended to other cohorts. Conversely, the incorporation of evidence significantly enhanced the significance of L-RoB trials, particularly those characterised by a substantial sample size, robust multivariate analysis, and comprehensive multi-point weight loss tracking.

The comprehensive application of the NOS tool, Newcastle–Ottawa Scale for non-randomized studies, following the 2011 updated version as recommended by the Cochrane Handbook for Systematic Reviews of Interventions, provided a robust method for assessing the reliability of gene-WL interactions post-RYGB. This systematic review was conducted to minimise selection, confounding, and measurement biases in the data utilised. Examining the impact of changes in *MC4R*, *FTO*, *LEPR*, *SH2B1*, and *POMC* on RYGB outcomes via the CNS–LEP–MC–INS–BA axis is also crucial.

Due to the significant variability across the included studies, particularly regarding outcome definitions, genetic models, follow-up durations, and effect estimates, a quantitative synthesis (meta-analysis) was not conducted. A qualitative narrative synthesis of the results was conducted instead. This demonstrated the association between specific gene variants and weight loss during RYGB. This study, being a systematic review of already published data and lacking direct patient interaction or identifiable personal information, did not require ethical approval or informed consent. AI-assisted language editing was the exclusive method for enhancing grammar and style. The tool did not generate content, assess data, or conduct scientific research.

Two groups of endpoints were predetermined. Reproductive endpoints include ovulatory function, menstrual regularity, anti-Müllerian hormone (AMH), reproductive hormones (FSH, LH, and oestradiol), and, where applicable, time-to-pregnancy or IVF indices. Weight gain, total body weight loss (TBWL), percentage of excess weight loss (%EWL), change in body mass index (ΔBMI), and glycemic/metabolic indices exemplify metabolic goals. No numerical synthesis of reproductive outcomes exists owing to the sporadic reporting of reproductive endpoints in the relevant literature, which were not provided in a genotype-stratified, variance-qualified style.

A formal meta-analysis was not conducted due to inadequate alignment of study outcomes, follow-up intervals, surgical techniques, genetic coding, and variance reporting.

We followed the PRISMA 2020 guidelines for identification, screening, checking eligibility, and inclusion. All records that were gathered from different places were deduplicated before being put through a reference manager. Two independent reviewers conducted the screening process in two stages: (i) reviewing titles and abstracts against predetermined eligibility criteria, and (ii) reviewing full-text reports that may be eligible. To ensure that the criteria were applied consistently, reviewers made decisions on a pilot set before the formal screening. Disputes were resolved at both stages initially through discussion and subsequently, if required, through adjudication by a third reviewer. There was a screening log that was set up ahead of time.

All reports stipulated identical criteria for eligibility to apply. To guarantee transparency, full-text exclusions were documented using defined categories and a single major rationale for each study: Insufficient data (e.g., absence of effect estimates or variance metrics hindering interpretation), ineligible design (e.g., reviews, editorials, protocols, case reports), duplicate or overlapping cohort (superseded by a more comprehensive publication), non-genetic or absent variant data; genotype-stratified outcomes, incorrect or irrelevant endpoints (not aligned with pre-specified outcomes) and incorrect population or procedure (e.g., non-bariatric or mixed procedures failing to meet inclusion criteria). The PRISMA flow diagram illustrates the quantities for each exclusion group. Upon polite request, we may provide the screening record that elucidates each choice.

Two reviewers used a structured form similar to the eligibility framework (population, technique, gene/variant, outcome definitions, follow-up window, and analytic model) to independently extract data post-inclusion. A third reviewer was used to resolve disputes and achieve consensus where necessary. We annotated reports with non-standard endpoint definitions or ambiguous timepoints and documented any deviations from the expected results to ensure these constraints were included in the synthesis.

To generate a unified summary effect, robust harmonisation assumptions would have been requisite, posing considerable risk due to the inclusion of studies employing disparate outcome metrics (such as TBWL versus %EWL versus composite glycaemic indices), misaligned follow-up periods, varied surgical modalities, and inconsistent genetic models/codings frequently lacking variance-qualified estimates. Therefore, as detailed in the Section 2, we provide a distinct qualitative/semiquantitative synthesis rather than performing a formal meta-analysis.

## 3. Results

### 3.1. Study Selection and PRISMA Flow

A comprehensive Boolean search conducted on PubMed, Scopus, and Web of Science yielded 2147 records across all three databases. After eliminating 612 duplicates, 1535 documents remained for title and abstract verification. Of these, 1411 studies were excluded for failing to conform to the established PICO framework or lacking genetic analysis related to bariatric surgery outcomes.

**Figure 1:**
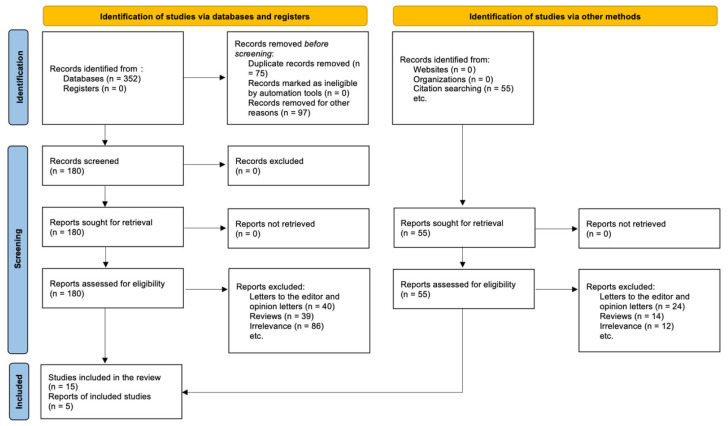
Prisma Flow Chart.

The remaining 124 items were assessed for full text eligibility. Ultimately, 20 publications fulfilled all inclusion criteria and were incorporated into the final qualitative synthesis. The primary reasons for the exclusion of full-text were: failure to provide outcomes by genotype, inclusion of surgical techniques other than RYGB without subgroup analysis, and insufficient follow-up duration (<6 months). Figure 1 illustrates the complete procedure of study selection as depicted in a PRISMA 2020 flow diagram.

### 3.2. Study Characteristics

This systematic review comprises 20 studies conducted between 2011 and 2022, involving participant groups from several regions, including the EU, LA, and NA. The research examined almost 5000 patients who underwent RYGB, with the majority of the cohorts predominantly consisting of women. The designs encompassed PC, RC, and NCC, facilitating the longitudinal tracking of weight fluctuations post-RYGB and their categorisation into groups. The follow-up durations varied significantly, spanning from 6 months to over 60 months. This enabled the examination of both WL and WR phenotypes throughout time.

All studies primarily concentrated on examining SNPs located inside genes that are functionally associated with significant metabolic and neuroendocrine pathways. FTO, MC4R, BDNF, NPY, and GHSR were all prominent targets. All of these factors are crucial for signalling inside the hypothalamic ARC-PVN. FTO SNPs, particularly rs9939609, have been extensively researched due to their influence on hypothalamic energy sensing, ghrelin control, and dopaminergic activity. Researchers examined MC4R polymorphisms, frequently located in the 3′ UTR (e.g., rs17782313), as they participate in α-MSH-mediated activation of cAMP signalling via Gαs coupling.

Several studies examined components of the leptin axis, including *LEP*, *LEPR*, and the subsequent phosphorylation cascades of STAT3. We examined various *LEPR* variants, such as Q223R, to assess their impact on SOCS3-mediated feedback inhibition and central leptin resistance. Both factors may alter the body’s adaptation to surgery. We investigated other SNPs associated with leptin to determine their correlation with %EWL, %TBWL, and WR, particularly concerning signalling in adipocytes and the integration of responses in the hypothalamus.

Polymorphisms in IRS1, IGF1, and ADIPOQ were utilised to investigate peripheral energy sensing, indicating alterations in the PI3K-AKT and AMPK pathways following RYGB. Alterations in IRS1 SNPs (including rs2943641) were associated with modifications in insulin sensitivity and GLUT4 translocation, whereas differences in IGF1 influenced feedback to the HPA axis, subsequently affecting GH secretion and IGFBP-bound hormone reservoirs. We examined ADIPOQ SNPs to determine their influence on the synthesis of adiponectin receptors and the activation of the PPARα pathway, both of which are crucial for enhancing insulin sensitivity and promoting fat oxidation post-surgery.

Novel pathways encompassed bile acid signalling genes such as FXR, TGR5, and SHP. This indicates an increased interest in enterohepatic feedback subsequent to RYGB. Variations in FXR, such as rs56163822, influence the secretion of FGF19, the composition of the bile acid pool, and the release of GLP-1. Researchers examined the importance of TGR5 SNPs in stimulating cAMP-PKA in intestinal L-cells, subsequently leading to insulin release via incretin. Certain studies also examined SHP and FGF21, which are associated with mitochondrial biogenesis and lipid metabolism in the liver.

The genotyping methods employed were distinct. The predominant methods employed were TaqMan-based qPCR assays, however certain studies utilised PCR-RFLP, Sanger sequencing, or NGS platforms. Analyses were predominantly conducted utilising dominant, recessive, or additive models. Numerous studies have also conducted subgroup analyses depending on allele dosage. Hardy–Weinberg equilibrium was seen in the majority of instances, though not universally.

The definitions of outcomes exhibited variability. The predominant metric discussed was %EWL, succeeded by %TBWL and absolute ΔBMI. Various studies defined weight recovery (WR) differently, with some indicating it as a recovery of ≥10% from nadir and others as ≥25% of total weight lost. There was a paucity of literature examining binary outcomes such as DM2R, HTNR, or OSA remission. Nonetheless, multiple studies examined biochemical outcomes such as ΔHbA1c or ΔHOMA-IR.

The included research examined various molecular factors across the CNS, HSA, INS, BA, and ADI axes. This indicates that WL and WR phenotypes following RYGB are polygenic. Table 3 provides a comprehensive summary of each study’s country, design, sample size, genotyped SNPs, involved pathways, genotyping method, follow-up duration, and primary outcomes.

Table 3 presents significant research examining the impact of genetic variations in *FTO*, *MC4R*, *LEP*, *LEPR*, and *POMC* on weight alterations following bariatric surgery. Each of these genes encodes crucial regulators of energy equilibrium, neuroendocrine appetite regulation, and signalling for adipose storage. Their many polymorphic forms influence the efficacy of bariatric therapies by engaging distinct biological pathways.

The *FTO* gene, particularly the intronic mutation rs9939609, has emerged as a primary focus for numerous research groups. This SNP resides in a non-coding region but functions as a long-range enhancer, particularly influencing the promoters of IRX3 and IRX5 in the hypothalamus and adipose tissue. These genes diminish mitochondrial thermogenesis by promoting white adipocyte identity instead of beige thermogenic transformation. Individuals possessing two copies of the A gene exhibit reduced capacity for browning and an increased propensity for energy storage. Post-surgery, catabolic conditions initially impede this mechanism, as indicated in early follow-up studies; nevertheless, this disparity diminishes over time, consistent with the epigenetic plasticity of IRX loci. The delayed convergence indicates that the FTO effect may not pertain to rapid weight loss, but rather to the capacity to resist retraining the body’s energy expenditure over time [43,46].

The *MC4R* genes in the paraventricular nucleus of the hypothalamus respond to α-MSH derived from POMC cleavage. Its signalling stimulates adenylate cyclase via Gαs, resulting in elevated cAMP levels that initiate thermogenic reactions subsequently. Variants such as rs17782313, I251L, and R165W exhibit distinct functional effects. The I251L mutation enhances receptor functionality by maintaining consistent ligand affinity and G protein coupling. This facilitates lipolysis and reduces hunger post-surgery [50]. Conversely, loss-of-function mutations such as R165W inhibit ligand-induced conformational alterations or impede cellular motility on their surfaces. This diminishes MC4R activity despite elevated leptin levels and reduced ghrelin, a condition observed post-bariatric surgery. These issues are exacerbated with LSG due to the lesser alteration of the gut–brain axis compared to RYGB. Campos et al. and Gong et al. demonstrate that these polymorphisms result in premature plateauing and weight increase following weight loss, so indicating a mechanistic role in central leptin-melanocortin resistance that persists despite alterations in gut shape [17,55].

*LEPR* polymorphisms, such as rs1137101 (Q223R), hold functional significance as they reside within the receptor’s extracellular or transmembrane domains. Q223R alters ligand binding affinity to receptors and the rate of their recycling. Leptin binding to LEPR activates JAK2, which phosphorylates STAT3. This facilitates the translocation of the transcription factor to the nucleus, where it regulates the expression of neuropeptides such as *POMC* and *CART*. Q223R mutations disrupt STAT3 signalling, inhibiting the negative feedback mechanism of SOCS3. This perpetuates leptin resistance even subsequent to significant fat mass reduction. Despite significant reductions in leptin levels post-surgery, Q223R carriers may exhibit diminished sensitivity to the hormone, perhaps resulting in decreased appetite and elucidating why certain populations experience lesser weight loss or regain weight more rapidly [47,56]. The discrepancies among studies may stem from differences in peripheral leptin dynamics, oestrogen modulation, and inflammation, all of which could influence the precision of the JAK2-STAT3 signalling pathway.

Variations in the *LEP* gene, such as rs7799039 (−2548 G>A), are located in the promoter region and influence the transcription of leptin mRNA. The A allele reduces leptin availability, hence diminishing its efficacy in appetite suppression inside the brain. In bariatric surgery, where caloric intake is significantly diminished and adipose leptin synthesis declines, individuals with *LEP* variations exhibiting reduced transcriptional activity may have an uncontrolled alteration in energy balance, particularly during the refeeding and maintenance stages. However, it is challenging to ascertain the precise impact of *LEP* polymorphisms due to the overlapping effects of *LEPR* sensitivity and downstream signalling.

*POMC* constitutes a significant component of this regulatory axis. Upon processing by *PCSK1*, it converts into α-MSH. Alterations in the *POMC* coding regions, such as frameshift or missense mutations, can disrupt peptide processing or receptor binding. This can inhibit melanocortin signals from functioning. Li et al. and Cooiman et al. both indicated that individuals with these mutations do not achieve the expected weight loss, particularly after restrictive procedures [48,49]. This is logical from a molecular perspective, as insufficient α-MSH synthesis would restrict MC4R activation, hence diminishing the thermogenic and anorexigenic responses necessary for continued weight loss. The failure to elevate *CART*, which collaborates with *POMC* to inhibit orexigenic NPY/AgRP neurones, exacerbates hyperphagic drive during metabolic adaption post-surgery.

A comprehensive long-term follow-up was conducted by Campos et al. They observed individuals for over 15 years and determined that those possessing heterozygous variants in these genes consistently face the risk of weight regain [17]. The interplay of partial issues related to FTO-driven thermogenesis, *LEPR*-mediated leptin signalling, and MC4R responsiveness complicates weight loss and maintenance. Initially, these patients may exhibit a favourable response to the physiological and hormonal alterations induced by surgery; nonetheless, they may ultimately succumb to their inherent neuroendocrine disorders. The arcuate nucleus contains critical signalling circuits, including AMPK, mTORC1, and PI3K/Akt, which are activated by leptin and insulin. These circuits were not directly investigated, yet they likely contribute to individual differences.

The findings in Table 3 indicate that surgical outcomes in bariatric patients are influenced not only by physiological and behavioural alterations but also by genetic variations that impact their metabolic responses to meals. These polymorphisms alter the efficacy of hypothalamic networks in facilitating catabolic signalling, modify the interaction between hormones from the stomach and brain, and determine an individual’s weight gain or loss. The varied responses to procedures such as RYGB and LSG further underscore the necessity of basing surgical planning on hereditary factors. In the future, incorporating genotyping into clinical pathways may identify individuals at elevated risk for adverse long-term outcomes and facilitate the planning of customised follow-up and supplementary treatments.

### 3.3. Genetic Associations with Postoperative Weight Loss

An analysis of the 20 trials revealed significant variations in the influence of genetics on weight loss following bariatric surgery. The *FTO* gene, particularly the intronic variant rs9939609, has emerged as the most extensively researched locus due to its established influence on obesity susceptibility and energy homeostasis regulation. The A allele, present in the AA and AT genotypes, is associated with increased body fat, reduced thermogenesis, and heightened energy intake, mostly due to its disruption of hypothalamic function and the commitment of adipocytes to their predetermined state. The review encompassed research by De Luis et al., Balasar et al., and Rodrigues et al., which demonstrated that those with the AA or AT genotypes experienced much less weight loss in the initial weeks post-surgery compared to those with the TT homozygote genotype. This discovery aligns with our understanding of the A allele’s influence on the expression of distant genes, such as IRX3 and IRX5, which inhibit mitochondrial oxidative phosphorylation and the browning of white adipose tissue, thereby reducing resting energy expenditure [43,45,46].

The *FTO* risk allele significantly influences outcomes in the initial months post-surgery, however this effect appears to diminish over time. Multiple studies indicated that the weight reduction trajectories of various genotype groups begin to converge by the 9- to 12-month follow-up period. This indicates that the abrupt alterations in metabolism and hormonal levels resulting after bariatric surgery—such as elevated GLP-1, diminished ghrelin, and reduced leptin—may temporarily surpass hereditary predispositions. Conversely, Rodrigues et al.’s study highlighted a crucial observation: after this key period, those possessing the A allele had a greater propensity for weight regain. This indicates that genetically designed homeostatic systems reactivate following the conclusion of the medically induced catabolic state [45].

At the molecular level, the *FTO* variant does not directly alter the protein’s demethylase function. Rather, it modifies the methylation of m^6A RNA, hence affecting the stability and splicing of transcripts associated with thermogenesis, insulin signalling, and lipid oxidation. This may impede AMPK functionality, diminish UCP1 levels in adipose tissue, and disrupt leptin receptor feedback mechanisms, so attenuating the metabolic benefits of weight loss. Furthermore, hypothalamic circuits including AgRP/NPY neurones, which exhibit heightened activity in individuals possessing the *FTO* A gene, may progressively re-establish control as nutrient consumption normalises post-surgery. This may result in heightened hunger and a resurgence in fat accumulation.

These results underscore the significance of long-term follow-up with patients possessing risk alleles in *FTO*. Early weight loss following surgery may not indicate that the metabolic programming is enduring. The convergence of weight trajectories at one year should not be interpreted as identical outcomes; rather, it indicates that the procedure induced a temporary hormonal equalisation that may not last without lifestyle modifications or additional treatment. Genotype-by-time interaction models may more effectively predict postoperative outcomes and identify individuals who may benefit from enhanced surveillance, dietary support, or pharmacogenetic interventions in the long term.

The *MC4R* gene, responsible for encoding the melanocortin-4 receptor, was identified as a significant genetic determinant of weight loss in patients post-surgery, particularly in those undergoing RYGB. This G-protein-coupled receptor is essential for maintaining equilibrium in the arcuate nucleus of the hypothalamus regarding energy regulation. This is the final stage in the *leptin–POMC–MC4R* pathway. The included research frequently examined variations such as rs17782313, I251L, C277X, and R165W, as they are significant for regulating appetite, sympathetic outflow, and thermogenic tone.

Individuals possessing the I251L variant, characterised as a gain-of-function mutation, consistently exhibited greater weight loss following bariatric surgery. This mutation enhances receptor sensitivity to endogenous agonists such as α-MSH. This enhances the activity of downstream effectors such as cAMP–PKA and CREB, which transmit signals that reduce hunger and increase caloric expenditure. Conversely, individuals with deleterious mutations such as R165W or C277X have diminished melanocortin signalling, resulting in less effective central satiety signalling, impaired regulation of sympathetic tone, and increased difficulty in adapting to energy deficits. Individuals often experience a slower initial weight loss and a more rapid regain, particularly following restrictive procedures such as LSG, where the neuroendocrine adaptation of the gut–brain axis is less significant compared to RYGB.

Numerous significant studies systematically examined the consequences of these MC4R polymorphisms. Gong et al. discovered that those with the R165W and G233S variants experienced a lesser degree of weight loss following either RYGB or SG procedures [55]. This was likely due to excessive caloric intake coupled with insufficient caloric expenditure. Mirshahi et al. discovered that individuals with the I251L variant had improved metabolic alterations and weight reduction over time, so reinforcing the notion that this variant may preserve or enhance hypothalamic reactivity post-surgery [50]. Resende et al. discovered that individuals possessing the rs17782313 polymorphism were more inclined to maintain a BMI above 30 kg/m^2^, indicating prolonged overweight status [54].

When MC4R malfunctions, it disrupts the hypothalamus’s capacity to transmit leptin and insulin signals reciprocally. Typically, these signals converge via α-MSH generated from POMC neurones to activate MC4R and inhibit AgRP/NPY neuronal activity. Loss-of-function variants maintain an orexigenic propensity, counteracting the effects of bariatric operations that often induce satiety and enhance calorie expenditure. These alterations may also diminish BAT activation and UCP1 expression, hence reducing the efficacy of adaptive thermogenesis. Furthermore, as MC4R is present in the autonomic nervous system, mutations may influence sympathetic stimulation of peripheral tissues, hence reducing lipolysis and glucose homeostasis.

The *MC4R* gene, responsible for encoding the melanocortin-4 receptor, was identified as a significant genetic determinant of weight loss in patients post-surgery, particularly among those who underwent RYGB. This G-protein-coupled receptor is essential for maintaining equilibrium in the arcuate nucleus of the hypothalamus regarding energy regulation. This represents the final stage in the *leptin–POMC–MC4R* pathway. The included research frequently examined variations such as rs17782313, I251L, C277X, and R165W, as they are significant for regulating appetite, sympathetic outflow, and thermogenic tone.

Individuals possessing the I251L variant, a gain-of-function mutation, consistently exhibited greater weight loss following bariatric surgery. This mutation enhances receptor sensitivity to endogenous agonists such as α-MSH. This enhances the activity of downstream effectors such as cAMP–PKA and CREB, which transmit signals that reduce hunger and increase caloric expenditure. Conversely, individuals with deleterious mutations such as R165W or C277X have diminished melanocortin signalling, resulting in less effective central satiety signalling, impaired regulation of sympathetic tone, and increased difficulty in adapting to energy deficits. Individuals often experience a slower initial weight loss and a more rapid regain, particularly following restrictive procedures such as LSG, where the neuroendocrine adaptation of the gut–brain axis is less significant compared to RYGB.

Numerous significant studies systematically examined the consequences of these MC4R polymorphisms. Gong et al. discovered that individuals with the R165W and G233S variants have less weight loss following either RYGB or SG procedures. This was likely due to excessive caloric intake coupled with insufficient caloric expenditure [55]. Mirshahi et al. discovered that individuals possessing the I251L variant had improved metabolic alterations and weight reduction over time, so reinforcing the hypothesis that this variant may preserve or enhance hypothalamic reactivity post-surgery [50]. Resende et al. discovered that individuals possessing the rs17782313 polymorphism were more inclined to maintain a BMI above 30 kg/m^2^, indicating prolonged overweight status [54].

When *MC4R* malfunctions, it disrupts the hypothalamus’s capacity to transmit leptin and insulin signals reciprocally. Typically, these signals converge via α-MSH generated from *POMC* neurones to activate *MC4R* and inhibit AgRP/NPY neuronal activity. Loss-of-function variants maintain an orexigenic propensity, counteracting the effects of bariatric operations that often induce satiety and enhance calorie expenditure. These alterations may also diminish BAT activation and *UCP1* expression, so rendering adaptive thermogenesis less efficacious. Moreover, since *MC4R* is present in the autonomic nervous system, mutations could influence the sympathetic stimulation of peripheral tissues, resulting in diminished lipolysis and glucose homeostasis.

Variants in the *LEPR* gene, particularly rs1137101 (Q223R), have garnered significant attention due to their potential impact on weight loss outcomes following bariatric surgery. This non-synonymous SNP alters glutamine to arginine at position 223 in the extracellular domain of the long-form leptin receptor (LEPRb), which is the primary mechanism by which leptin signals in hypothalamic neurones. It has been proposed that the Q223R polymorphism may influence ligand-receptor binding affinity, receptor internalisation efficiency, and the signal transduction efficacy of the JAK2–STAT3 pathway. The primary function of leptin is to reduce hunger and enhance energy expenditure.

This review examined studies by Kops et al. and Novais et al. that indicated individuals with the AA genotype (Q/Q) would experience more weight loss following RYGB [47,56]. This is likely due to their heightened sensitivity to *LEP* and the continued suppression of their hypothalamic–pituitary–adrenal (HPA) axis post-surgery. Q/Q carriers may exhibit enhanced leptin signal fidelity, perhaps resulting in increased STAT3 phosphorylation and elevated levels of *POMC*, *SOCS3*, and *CART.* These three hormones collaborate to diminish orexigenic tone and enhance catabolic neurocircuitry. The functional effects of Q223R remain contentious, as other studies included did not establish the same affirmative correlation. This may result from gene–environment interactions or the impact of cytokine-induced leptin resistance, particularly in those with elevated CRP or systemic low-grade inflammation.

We also examined the impact of the LEP promoter mutation rs7799039 (−2548 G/A) on weight alterations. This SNP may alter the transcription of the *LEP* gene, hence affecting the levels of leptin in the bloodstream in relation to fluctuations in adipocyte mass and energy homeostasis. Certain studies indicated that individuals with AA might exhibit elevated leptin levels at rest, potentially resulting in less hunger post-surgery. This impact did not appear consistently, perhaps due to the non-linear nature of leptin-adiposity feedback loops, resulting in a more significant decrease in leptin levels than in fat mass reduction, a phenomenon termed “relative hypoleptinemia.”. In this instance, alterations in *LEP* genotypes may be of minimal significance, as the surgical suppression of insulin, ghrelin, and pro-inflammatory cytokines predominantly regulates energy balance in the initial days post-surgery.

Furthermore, leptin signalling operates in conjunction with other mechanisms. The interplay of AMPK, mTOR, and IRS pathways alters the hypothalamic response and influences outcomes. LEPR mutations may alter this equilibrium; however, their phenotypic manifestation may only occur in specific endocrine or inflammatory contexts, such as PCOS, T2DM, or chronic hyperinsulinemia. The varying impacts of *LEP/LEPR* polymorphisms on distinct tissues, such as white adipose tissue, brown adipose tissue, and liver, may elucidate the differential weight loss outcomes observed post-bariatric surgery.

Research conducted by Li et al. and Cooiman et al. has demonstrated that *POMC* polymorphisms significantly influence weight regulation post-bariatric surgery. Individuals with missense or nonsense variations experienced more difficulty in maintaining weight loss post-surgery [48,49]. These mutations likely disrupt the conversion of pro-opiomelanocortin into bioactive peptides post-translation, particularly α-MSH, which is essential for MC4R activation. Due to α-MSH’s mechanism of action via the cAMP–PKA–CREB pathway to diminish orexigenic stimulation, any reduction in its bioavailability renders hypothalamic satiety signals less efficacious. The subsequent reduction in melanocortin tone facilitates rebound hyperphagia and diminishes lipolytic drive, particularly as compensatory neurohormonal feedback mechanisms reinitiate throughout the mid to late postoperative phase.

The longitudinal results from Campos et al. corroborated these mechanistic disturbances. They monitored patients for a duration of up to 15 years and demonstrated that heterozygous carriers of mutations in the *LEP–LEPR–POMC–MC4R* axis consistently struggled to maintain weight loss [17]. The results indicate that minor disruptions in anorexigenic signalling can significantly alter energy balance homeostasis, potentially due to the hypothalamus’s inability to accurately recalibrate its set-point. Furthermore, these polymorphisms may impede the adaptability of hypothalamic neuronal circuits to alterations in gut-derived peptide flux following surgery. This is particularly applicable to peptides such as GLP-1, PYY, and oxyntomodulin, which often enhance the activity of POMC neurones.

Numerous investigations indicated that gene–procedure relationships were significant. The impact of *FTO* and *MC4R* mutations was consistently more pronounced in those who had LSG, a purely restricted method that excludes foregut exclusion and biliopancreatic rearrangement found in RYGB. RYGB may partially circumvent upstream hypothalamic dysfunction, particularly in the early postoperative phase, by enhancing enteroendocrine signalling and inducing significant alterations in gut–brain communication. Conversely, LSG depends more on internal central mechanisms, facilitating the identification of issues related to leptin–melanocortin signalling, NPY/AgRP inhibition, and the autonomic regulation of fat metabolism.

This unique reaction to surgery underscores the need of neurogenetic stratification for those seeking weight loss. Individuals with biallelic or functionally significant changes in POMC or its pathway components may derive greater benefits from RYGB-like interventions. Conversely, restrictive methods may prove insufficient to overcome central leptin resistance or melanocortin insensitivity. In the future, the integration of SNP screening, mRNA expression profiling, and functional hormone testing may enable the customisation of surgical procedures to align with the underlying molecular problem, ensuring that the physical intervention corresponds to the patient’s metabolic genotype.

Direct reproductive outcomes were seldom recorded and, when present, lacked genotype differentiation or variation assessment. As a result, no eligible datasets allowed per-gene quantitative summary for ovulatory function, menstrual regularity, LH/FSH/estradiol levels, AMH, or time-to-pregnancy/IVF metrics.

Our title highlights genes related to fertility, since leptin–melanocortin biology connects energy balance to reproductive function. We refrain from providing numerical data on reproductive outcomes to avoid misunderstanding, given metabolic endpoints mostly characterise the existing evidence post-bariatric surgery, with little genotype-stratified reproductive metrics available. Future research must provide genotype-stratified, variance-adjusted estimates and establish standardised reproductive endpoints for comparative synthesis.

## 4. Discussion

Our research clarifies the influence of leptin melanocortin changes on postoperative results in bariatric surgery by combining pathway biology with a semi-quantitative examination of research methodologies. When melanocortin tone is likely maintained (as in MC4R settings), signals demonstrate optimal coherence across various designs. This corresponds with improved weight loss or more favourable metabolic pathways in more uniform situations (kind of treatment and follow-up). Despite the uneven effects of FTO, which vary according to behavioural and surgical situations, studies concerning *LEP*/*LEPR* and *POMC* indicate minor, context-dependent affects associated with hunger and energy expenditure pathways. We refrain from making quantitative claims on reproductive recovery owing to the limited availability of genotype-stratified data in the relevant literature. However, the molecular connection between energy homeostasis and hypothalamic–pituitary–gonadal signalling constitutes a physiologically credible route that needs direct examination in future research. This systematic analysis included 20 studies investigating the association between genetic variations and weight loss following bariatric surgery. The results indicated significant variability across the studies regarding their methodology, duration of follow-up, and the specific genes examined. Nonetheless, distinct patterns indicated the significance of genetic loci in influencing weight reduction.

The *FTO* gene, particularly the rs9939609 polymorphism, was the most extensively studied. It was associated with decreased weight loss in the initial phases and an increased likelihood of weight regain over time, particularly in individuals possessing the A allele. Various polymorphisms in MC4R, including rs17782313, I251L, and R165W, exhibited distinct effects based on their mechanisms of action. For instance, loss-of-function variations rendered restricted approaches such as LSG less efficacious.

The discoveries about the LEPR and LEP genes were equally significant. Variants such as rs1137101 and rs7799039 altered leptin sensitivity and its signalling in the hypothalamus. Mutations in *POMC*, particularly those impacting the synthesis of α-MSH, have been associated with reduced weight loss and expedited weight regain. This illustrates the significance of the leptin–melanocortin axis in energy regulation post-surgery.

A relationship is deemed statistically validated when a directionally analogous signal is found under more consistent settings (aligned outcomes, follow-up, and methods) and/or is duplicated across diverse cohorts using variance-qualified estimates. Results that fail to meet these requirements are deemed hypothesis-generating, shaped by pathway priors (e.g., lower melanocortin signalling predicting less weight loss). This differentiation facilitates the identification of areas with insufficient evidence (e.g., genotype-stratified reproductive endpoints) and sets priorities for validation targets (e.g., *MC4R* contexts demonstrating convergent directions), while preventing the conflation of biological plausibility with empirical validation.

Ultimately, several investigations demonstrated that the consequences of these SNPs varied according to the type of bariatric surgery performed. For instance, malabsorptive treatments such as RYGB largely compensated for preexisting neuroendocrine problems. The synthesis underscores the significance of examining the impact of various genotypes on metabolic responses to bariatric surgery. It further endorses the emerging concept that obesity treatment should be founded on precision medicine.

### 4.1. Genetic Factors Influencing Post-Bariatric Surgery Weight Loss

This systematic investigation demonstrates the significance of genetic variation in influencing individual responses to bariatric surgery, particularly through alterations in the neuroendocrine pathways that regulate appetite, satiety, and energy expenditure. The *FTO* gene was the most extensively researched gene, with rs9939609 being the primary SNP of focus. Investigations conducted by De Luis, Balasar, Rodrigues, Kops, Bandstein, and Novais revealed that individuals possessing the A allele (AA or AT genotypes) experienced much less weight loss during the early postoperative phase compared to those with the TT genotype [13,43,45,46,47,56]. The role of *FTO* as a nucleic acid demethylase is responsible for this action. It primarily regulates the N6-methyladenosine (m6A) modifications to mRNA transcripts that are crucial for energy sensing in the hypothalamus [57]. Altered demethylation profiles result in the improper functioning of downstream effectors such as IRX3 and IRX5. This alters the differentiation of adipocyte lineage towards energy-storing white adipose tissue rather than thermogenic beige or brown fat [58]. Consequently, individuals possessing the A allele have reduced baseline energy expenditure, less mitochondrial uncoupling, and weaker adaptive thermogenesis when consuming fewer calories. Rodrigues’s longitudinal data indicated that these epigenetic alterations may not only influence initial weight reduction but may also predispose individuals to metabolic adaptation and subsequent weight regain after two years post-surgery. This is likely due to the hypothalamus’s diminished responsiveness to leptin and alterations in the feedback regulation within the arcuate nucleus [45].

The MC4R gene, a crucial component of the central melanocortin system, garnered significant interest concurrently. α-MSH, a cleavage product of *POMC*, activates this GPCR in the paraventricular nucleus of the hypothalamus [59]. Gong, Mirshahi, Resende, Valette, Aslan, and Salazar-Valencia investigated polymorphisms such as rs17782313, I251L, R165W, and C277X. The I251L variant, a gain-of-function mutation, is believed to enhance Gαs-coupling, elevate cAMP production, and amplify anorexigenic signalling via CREB-dependent transcriptional mechanisms [5,7,50,52,54,55]. Mirshahi and Gong discovered that I251L carriers experienced greater weight loss and maintained it for an extended duration. This aligns with established knowledge on heightened sympathetic output, elevated energy expenditure, and diminished orexigenic neuropeptides such as AgRP and NPY. Conversely, the R165W and C277X variants are associated with deficiencies in receptor expression or ligand binding, resulting in a diminished anorexigenic response and reduced satiety signalling [50]. Resende indicated that individuals possessing the rs17782313 mutation were more prone to failing to achieve their BMI reduction objectives post-RYGB and frequently maintained BMI values over 35. This indicates that MC4R loss-of-function mutations impair the hypothalamus’s ability to interpret peripheral satiety cues, despite significant hormonal alterations in the gut [54].

The functional integrity of POMC was crucial for the surgical outcomes. Li, Cooiman, and Campos examined the impact of mutations on *POMC* cleavage and α-MSH bioavailability. Mutations in this gene can lead to the accumulation of non-functional intermediates or insufficient production of active melanocortins, as POMC undergoes a tightly regulated process following its translation by prohormone convertases such as PC1/3 and PC2 [48,49]. Li and Campos’s research shown that patients with nonsense or missense POMC mutations have difficulties in maintaining weight loss over an extended period and began to regain weight more rapidly [17,48]. Campos’s 15-year follow-up was particularly noteworthy as it demonstrated that dysfunctions in the *LEP–LEPR–POMC–MC4R* axis resulted in a sustained energy imbalance, despite alterations to the gastrointestinal tract’s anatomy [17]. Bariatric surgery may not fully resolve issues with neuroendocrine tone in the brain resulting from inadequate melanocortin signalling, despite significantly altering peripheral satiety signals [60].

Novais, Potoczna, and Kops examined the *LEP* and *LEPR* genes, which encode leptin and its receptor, respectively. Adipocytes secrete leptin according to their fat content. This interacts with *LEPR* on hypothalamic neurones and initiates JAK2–STAT3 phosphorylation cascades. These cascades inhibit AgRP/NPY neurones and facilitate POMC transcription. The rs1137101 polymorphism in *LEPR*, located in the extracellular domain, may impede ligand binding or signal transduction [47,51,53]. Kops indicated that those with the AA genotype experienced slightly greater weight loss post-surgery, potentially because to enhanced receptor functionality [47]. However, Novais and Potoczna observed no enduring alterations in energy consumption or weight results. This indicates that other routes, such as gut-derived hormones (GLP-1, PYY, and oxyntomodulin), may assume primary regulatory roles post-surgery, particularly following malabsorptive surgeries [51,56].

It is essential to acknowledge that multiple studies, like those conducted by Campos, Gong, and Potoczna, shown that the surgical approach influenced the impact of genetic variants on the phenotypic. In scenarios where central appetite regulation is paramount, for as in restrictive procedures like LSG or gastric banding, polymorphisms in *FTO*, *MC4R*, and *LEPR* significantly influenced outcomes [17,51,55]. Conversely, malabsorptive techniques such as RYGB or biliopancreatic diversion appear to mitigate the impact of these differences by broadly reprogramming metabolism, including enhancements in incretin signalling, alterations in bile acid profiles, and modifications in gut microbiota composition [61].

### 4.2. Integrating Recent Findings on the Molecular Genetics of Obesity with the Outcomes of Bariatric Surgery

Recent study has significantly enhanced our comprehension of the impact of genetic variations on the efficacy of MBS. This illustrates the intricate manner in which the CNS regulates hunger and energy equilibrium. The *LEP–LEPR–POMC–MC4R* axis is central to this regulatory network. It transmits data on peripheral adiposity to the hypothalamus, which then elicits neuroendocrine responses [62]. Alterations along this axis, including SNPs, epigenetic modifications, or rare monogenic variants, may disrupt satiety signalling, thermogenic drive, and autonomic control. This may diminish the long-term efficacy of MBS [63].

Fansa and Acosta stated that the hypothalamus melanocortin system is the primary mechanism via which leptin communicates the cessation of eating. LEP interacts with *LEPR* on neurones in the ARC, activating the STAT3 and PI3K pathways to enhance the transcription of *POMC.* PCSK1 subsequently cleaves *POMC* into α-MSH, which activates neurones expressing *MC4R* in the PVN. When functioning optimally, this system inhibits consumption by transmitting signals via CRH and TRH neurones and enhances sympathetic output to stimulate brown adipose tissue (BAT) thermogenesis and hepatic gluconeogenesis. Any instance involving a *LOF* mutation, such as *LEPR* Gln223Arg, POMC nonsense variations, or *MC4R* missense mutations, results in diminished α-MSH signalling, persistent activation of NPY/AgRP orexigenic neurones, and reduced SNS tone. These alterations not only diminish the satiety set-point but also reduce REE, complicating the ability of individuals with specific genotypes to undergo either restricted or malabsorptive surgery [64,65].

Poitou and his colleagues examined a cohort of individuals with bi-allelic pathogenic mutations in *LEPR*, *POMC*, or *MC4R* and discovered that they underwent RYGB or SG. Post-surgery, all patients initially had significant weight loss; however, they subsequently regained the weight, often exceeding their pre-surgical levels [66]. From a molecular perspective, *LEPR* mutations inhibit *LEP* from phosphorylating STAT3, hence preventing the transcriptional activation of *POMC*. *POMC* mutations inhibit prohormone convertase from cleaving the peptide, resulting in insufficient production of α-MSH peptides [67]. Mutations resulting in MC4R loss of function, particularly at the transmembrane or ligand-binding domains, render the receptor unresponsive to α-MSH, hence inhibiting the propagation of anorexigenic signals. These issues indicate that crucial downstream targets, such as the sympathetic stimulation of BAT, the secretion of GLP-1, and the mechanisms that enhance insulin sensitivity, are ineffective [68,69]. Poitou et al. stated that surgery alone is insufficient to surmount this genetically predetermined metabolic inertia without reinstating hypothalamic function [66].

Maculewicz et al. provided additional evidence from a cohort of guys who were not undergoing surgery. They examined SNPs in *FTO*, *LEP*, *LEPR*, *FABP2*, and *MC4R* in physically active men. Individuals possessing the *FTO rs9939609* A allele had significantly elevated FMI levels, despite maintaining a high level of physical activity. This mutation alters an intronic enhancer regulating the expression of IRX3 and IRX5 in preadipocytes, steering the differentiation process towards white adipogenesis and away from UCP1-expressing beige and brown adipocytes. The functional consequence is that the mitochondria exhibit less uncoupling ability and reduced heat production [70]. The *MC4R rs17782313* polymorphisms diminished satiety and altered lipid metabolism, underscoring the receptor’s significance in nutrition sensing. The additional risk associated with possessing several SNPs, particularly when integrating *FTO*, *MC4R*, and *FABP2*, increases the likelihood of developing metabolic syndrome, even in the absence of observable symptoms [6,71]. This indicates that the genetic burden can independently alter metabolic tone and energy distribution.

Kunnathodi et al. adopted a comprehensive perspective, elucidating how both prevalent and atypical mutations influence epigenetic and transcriptomic factors to produce various manifestations of obesity. The *FTO* gene has been shown to alter the methylation state of mRNA transcripts associated with oxidative phosphorylation and mitochondrial biogenesis. This epitranscriptomic alteration influences the efficiency of energy utilisation in skeletal muscle and may impact post-surgical energy expenditure [72]. *MC4R* polymorphisms were linked to impairments in BDNF-mediated neuronal plasticity in the VMH and PVN, critical regions for the long-term regulation of appetite and stress [73]. Furthermore, DNA methylation of the *LEP* and *LEPR* promoter regions exacerbates central leptin resistance, particularly in individuals who experienced inadequate nutrition prenatally or who suffer from chronic low-grade inflammation [74]. Kunnathodi et al. discussed the application of GRS and PRS in forecasting post-MBS outcomes. These scores amalgamate data from numerous loci. Individuals with elevated GRS profiles had reduced %EWL and experienced earlier rebound, indicating that a multi-omic categorisation approach may assist physicians in more effectively categorising patients and customising treatments for each individual [72].

The systematic review conducted by Zafirovska et al. is the most comprehensive to date. It examined 52 studies investigating the impact of genetic variants on MBS results. The scientists determined that *FTO* and *MC4R* SNPs are significant, particularly in distinguishing early from late weight loss patterns. *FTO* polymorphisms, such as rs9939609 and rs1421085, exerted a greater influence on SG recipients than on RYGB recipients [75]. This may be due to the gut–brain axis being less adaptable in regulating operations compared to RYGB. Loss-of-function mutations in *MC4R* were associated with less satiety postprandially and an increased propensity for weight regain, particularly in the absence of concurrent activation of the GLP-1 pathway [76,77]. Additional loci, including as GLP1R, PGC1A, and ADIPOQ, were associated with alterations in insulin sensitivity post-surgery, mitochondrial ATP production, and adipokine release [78]. For instance, various alleles of GLP1R were associated with alterations in incretin response, whereas distinct variants of PGC1A SNPs influenced mitochondrial function and oxygen utilisation. The *LEP223* and *LEPR Q223R* variants were particularly noteworthy as they altered the kinetics of ligand-receptor interaction and the activation of the JAK/STAT pathway, hence influencing the hypothalamic set point and the thresholds for energy storage [79].

These results demonstrate that polymorphisms in *FTO*, *MC4R*, *POMC*, and *LEPR* establish a complex molecular framework that alters both the short- and long-term outcomes of bariatric surgery. *FTO* variants diminish the flexibility of adipocytes and their capacity for thermogenesis. *MC4R* mutations disrupt the central satiety circuitry and sympathetic nervous system tone. *POMC* and *LEPR* deficits disrupt hormonal feedback and the transcriptional regulation of anorexigenic peptides. The alterations in the body’s physiology induce a neuroendocrine condition that inherently resists standard surgical interventions. This indicates that distinctive strategies are required, maybe encompassing CNS-acting pharmaceuticals such as setmelanotide, gene-targeted therapies, or modifications to surgical procedures.

### 4.3. The Clinical Utility of Genetic Screening Prior to Bariatric Surgery

Genetic screening has become an essential component in the planning of bariatric surgery due to the rapid advancement in our comprehension of the genetic underpinnings of obesity. This is a significant advancement towards the age of precision medicine [80,81]. Obesity was previously seen primarily as an environmental affliction; however, it is now understood to result from a complex interplay of genetic, epigenetic, neuroendocrine, and behavioural variables [82]. Increasing data indicates that SNPs and rare mutations in genes such as FTO, MC4R, LEPR, and POMC influence not only the risk of obesity but also significantly impact weight loss, WR, and metabolic outcomes following bariatric surgery [72]. Pereira et al. emphasised that bariatric surgery is effective; nevertheless, the long-term outcomes vary significantly across individuals, and these variations cannot be entirely attributed to the surgical process or the individual’s BMI before to surgery [80]. Conversely, variability in genetic backgrounds among individuals, particularly differences in central regulators of appetite and energy homeostasis, emerge as significant determinants influencing treatment responses. Incorporating genotypic data into preoperative evaluations may enhance the selection of optimal patients, surgical techniques, and prospects for long-term success, hence facilitating truly tailored procedures [83].

Poitou et al.’s retrospective observations constitute compelling data about the impact of rare genetic defects on bariatric outcomes. Both RYGB and SG resulted in weight reduction among patients with bi-allelic mutations in *LEPR*, *POMC*, or *MC4R.* Subsequently, nearly all patients had prolonged weight return, with a significant number recovering the majority of the weight they had previously lost within a few years. The failure arises from the molecular pathology of these genes: *LEPR* mutations inhibit leptin from activating STAT3 in arcuate nucleus neurones, thereby halting *POMC* transcription and α-MSH synthesis; POMC mutations prevent PCSK1 from cleaving prohormones, resulting in the absence of crucial melanocortin peptides; and MC4R loss-of-function variants render hypothalamic satiety circuits unresponsive to α-MSH signalling [66]. The ultimate outcome is a persistent desire to consume food, diminished SNS activity, and reduced REE. These are physiological problems that cannot be permanently resolved just through anatomical limitation or malabsorption. Zafirovska et al. conducted a systematic assessment of over 50 papers and confirmed the validity of these findings. Surgical outcomes are frequently transient in genetically predisposed individuals until supplemented by pharmaceutical interventions, such as *MC4R* agonists or GLP-1 receptor analogues, which directly influence central energy homeostasis [75].

Screening serves purposes beyond merely identifying individuals with severe monogenic obesity. Common polymorphisms, such as *FTO rs9939609* and *MC4R rs17782313*, have been linked to alterations in postoperative outcomes across extensive populations, although the magnitude of their impact differs [45,54]. Maculewicz et al. demonstrated that individuals possessing the *FTO* A allele, regardless of their weight or physical activity levels, exhibit an elevated FMI. *FTO* regulates the expression of thermogenic genes via IRX3/5 signalling. The allelic effects, when integrated with additional SNPs in *LEPR*, *FABP2*, or *PGC1A*, may impede post-surgical weight loss and increase the propensity for early weight gain by hindering mitochondrial proliferation, adipocyte browning, and lipid metabolism [70]. Van der Meer et al. examined SNPs in *FTO*, *MC4R*, *ADIPOQ*, and *UCP2.* They discovered that differences in ADIPOQ significantly influenced lipid and glucose levels post-surgery, while their association with weight loss was comparatively weaker. Polymorphisms in UCP2, particularly rs660339, were associated with diminished thermogenic responses post-surgery [84]. This indicates that the capacity to uncouple mitochondria is crucial for maintaining elevated energy expenditure post-bariatric surgery. The collective knowledge indicates that the genetic composition of obesity significantly influences both weight loss and the resolution of obesity-related health issues.

Pereira et al. assert that precision medicine in bariatric surgery must transcend the examination of individual genes and adopt a comprehensive predictive framework that incorporates genetic, metabolic, behavioural, and psychological factors. Age, sex, baseline BMI, and comorbidities such as type 2 diabetes are recognised as effective predictors of weight loss. Nonetheless, genetic background remains one of the least examined yet most intriguing variables [80]. Pereira et al. emphasise the necessity for machine learning algorithms that utilise preoperative characteristics such as genetic markers, circulating microRNAs, and metabolomic profiles to forecast which patients would successfully lose weight and maintain it, as well as those likely to regain it [80]. AI is advantageous as it can identify intricate, non-linear relationships among variables that conventional regression models are unable to detect. Incorporating *FTO* and *MC4R* polymorphisms into AI algorithms could assist physicians in identifying patients at elevated risk who may require intensified nutritional oversight or increased medication during the initial postoperative days [85]. This aligns with the findings of Van Der Meer et al. Candidate gene studies have not consistently yielded reliable results, and the absence of robust evidence for any single SNP does not imply that genetics are irrelevant to obesity; rather, it indicates that obesity is polygenic and necessitates high-resolution, multi-omic datasets [84].

From a translational perspective, the implementation of genetic screening in bariatric treatment could fundamentally transform clinical practices. Initially, validated gene panels targeting *FTO*, *MC4R*, *LEPR*, *POMC*, *ADIPOQ*, *PGC1A*, and *UCP2* will be incorporated into normal preoperative screens. This might assist physicians in identifying individuals who are less probable to respond to surgery alone. Surgical plans must be predicated on hereditary risk profiles. Patients with *MC4R* loss-of-function mutations may derive greater benefit from RYGB, which significantly influences gut–brain signalling and GLP-1 synthesis, compared to SG. Third, genotyping may facilitate the timely implementation of adjuvant pharmacological interventions, such as *MC4R* agonists or combination GLP-1/GIP therapies, to address issues related to energy regulation in the brain or body. Furthermore, genetic data may assist in predicting the resolution of comorbidities. *LEPR* variations may diminish leptin’s efficacy in reducing hepatic fat levels, while PGC1A polymorphisms may impede enhancements in skeletal muscle insulin sensitivity by compromising mitochondrial oxidative phosphorylation efficiency. Genetic forecasts may assist physicians in determining the necessity for intensified follow-up and further treatments post-surgery.

### 4.4. What Are the Forthcoming Developments in Precision Bariatric Medicine?

The advancement of bariatric surgery is rapidly aligning with the broader initiative of precision medicine. This strategy selects treatments based on the patient’s specific genetic, molecular, behavioural, and environmental context, rather than solely on broad phenotypic factors such as BMI. As our understanding of obesity’s multifactorial aetiology deepens, encompassing the interplay of central nervous system signalling, adipocyte function, alterations in gut hormones, and energy homeostasis, it becomes apparent that surgical intervention alone cannot fully enhance outcomes. This shift in perspective necessitates the comprehensive integration of -omics data, genotype-specific risk models, and long-term biomarker monitoring into the standard bariatric treatment protocol. This will enable the development of customised, anticipatory, and adaptive therapy approaches.

A primary objective of this modification is to develop and implement PRSs and validated gene panels in clinical settings to predict an individual’s response to specific surgical interventions. Numerous investigations, including those conducted by Zafirovska et al., van der Meer et al., and Maculewicz et al., have demonstrated that SNPs in genes such as *FTO*, *MC4R*, *LEPR*, *POMC*, *ADIPOQ*, and *UCP2* are associated with varying surgical results. Nonetheless, these outcomes must be included into composite prediction models [70,75,84]. PRSs that integrate the impacts of many SNPs can more effectively categorise individuals who are predisposed to weight loss, enhancement of blood glucose levels, and an increased likelihood of weight regain over time. Future research should aim to conduct similar evaluations utilising high-throughput genotyping across diverse populations, validate them against outcomes after 5 to 10 years, and assess their predictive efficacy for various surgical procedures, such as RYGB, SG, and emerging techniques like SADI-S or OAGB.

Multi-omic integration, encompassing transcriptomics, proteomics, epigenomics, metabolomics, and microbiomics, transcends static genetic profiles to offer a dynamic and systems-level perspective on the mechanisms of obesity. Alterations in FTO-mediated m6A RNA methylation may impede skeletal muscle’s ability to generate new mitochondria and metabolise lipids, hence hindering the body’s capacity to burn fat post-surgery [86]. Similarly, DNA methylation in *LEPR* promoter regions can diminish receptor expression, hence reducing the efficacy of leptin-mediated hypothalamic activation. Circulating miRNAs such as miR-122 and miR-33, which regulate lipid metabolism in the liver and thermogenesis in brown adipose tissue, may serve as biomarkers for predicting long-term metabolic outcomes before and after surgery [87]. Employing these molecular fingerprints in clinical decision-making, particularly through AI-driven algorithms, could enhance the precision of treatment planning and follow-up regimens. Pereira et al. assert that machine learning can discern intricate relationships among these variables, identify concealed anomalies, and provide risk maps applicable in clinical environments.

One of the most exhilarating prospects for the future is the adaptation of surgical methods to the specific pathophysiology of various genotypes. In patients with loss-of-function mutations in *MC4R* or *POMC*, where hypothalamic anorexigenic signalling is fundamentally impaired, interventions such as sleeve gastrectomy, which primarily depend on limited and moderate endocrine regulation, may prove insufficient. RYGB may be more advantageous for these individuals as it induces elevated secretion of GLP-1, PYY, and bile acids, compensating for issues with central pathways. Conversely, individuals possessing the *FTO* variant, who exhibit resistance to thermogenesis and modified adipose signalling, may benefit more from less invasive procedures if they subsequently administer pharmacological agents that stimulate brown adipose tissue via adrenergic or thyroid hormone pathways. These findings indicate that genotype-directed procedural algorithms ought to be developed. The selection of SG, RYGB, or combination therapies in these algorithms is grounded in scientific study aimed at rectifying molecular abnormalities, rather than conjecture.

An essential aspect of future care is the utilisation of adjunct medication informed by genotypic and transcriptomic data. Patients with CNS system mutations or reduced sensitivity to leptin may not fully benefit from mechanical restriction or dietary malabsorption alone. Consequently, they ought to be promptly examined for pharmacological agents that address their molecular anomaly. Setmelanotide is a selective MC4R agonist that has significantly affected individuals with *POMC*, *LEPR*, or *PCSK1* mutations. Similarly, GLP-1R agonists, dual incretin mimetics (GLP-1/GIP), and FGF21 analogues represent novel therapeutic alternatives for individuals with peripheral signalling disorders, facilitating increased caloric expenditure, appetite suppression, and enhanced hepatic sensitivity to insulin. Incorporating pharmacogenomics platforms into bariatric centres may assist in identifying patients who require early or preventive pharmacological intervention post-surgery to maintain weight reduction and avoid weight regain.

To employ accurate instruments in institutions, modifications in their structure and pedagogical approaches would be necessary. For the effective implementation of this paradigm, bariatric surgery clinics must integrate genomic counselling units, equip teams with the necessary instruments for SNP panel testing, and ensure the availability of bioinformatics support to interpret omics data. Clinical workflows must incorporate preoperative genetic stratification, intraoperative decision modification, and personalised postoperative monitoring. Longitudinal registries linking genotypic profiles with procedural outcomes will be crucial for feedback learning loops. Policymakers and relevant stakeholders should establish funding mechanisms for genetic testing, particularly for high-risk individuals, and enact regulations that delineate the standard of care for molecularly informed bariatric therapy.

Recent data indicates that this approach should be applied to adolescents and children, particularly those who are very obese at a young age and possess a familial predisposition to monogenic or syndromic causes. Early identification of patients with abnormalities in *LEPR*, *MC4R*, or *POMC* may provide prompt pharmacological intervention and defer surgical procedures until the body is adequately prepared. Genomic screening initiatives at educational institutions or primary healthcare environments could assist genetically predisposed children in implementing lifestyle modifications earlier, thereby preventing the onset of advanced metabolic diseases.

In summary, the future of bariatric surgery lies in its amalgamation with molecular medicine. The treatment constitutes a singular component of a broader therapeutic environment tailored to each individual’s biological composition. Surgeons and endocrinologists can identify the primary pathophysiological factors in each patient by utilising genetic, epigenetic, and transcriptomic data. They can thereafter select surgical or pharmacological interventions that are most suitable for the individual’s energy homeostasis profile. Precision bariatric treatment not only ensures superior outcomes but also guarantees care that is more ethical, efficient, and patient-centred. The subsequent stage is to actualise this vision by developing the tools, teams, and policies that will facilitate this molecular revolution in metabolic health.

The significant diversity in post-operative outcomes is likely due to non-genetic factors, even though our research concentrated on germline polymorphisms, especially within the leptin–melanocortin axis. Epigenetic mechanisms, including DNA methylation, histone changes, and microRNA-mediated post-transcriptional regulation, may alter the expression of *LEP/LEPR*, *POMC*, *MC4R*, and associated upstream/downstream nodes that govern hunger, thermogenesis, and insulin sensitivity. Bariatric surgery is associated with significant alterations in gut hormone levels, bile acid signalling, inflammation, and nutritional status. These modifications may provoke both temporary and permanent epigenomic changes in skeletal muscle, liver, adipose tissue, and haematopoietic cells. These alterations may intensify or diminish allelic effects on gene expression. They may also modulate pathway activity independently of genetics or, more likely, interact with genotype, such as via methylation at promoter/enhancer regions harbouring functional variations. This clarifies the reasons for the differing weight-loss and glycaemic improvement patterns seen in people with similar genotypes. To delineate these layers and improve risk stratification beyond genetic factors alone, paired genomic–epigenomic designs (baseline and 6–12 months post-surgery) that incorporate targeted methylation at leptin–melanocortin loci, chromatin accessibility, and circulating/adipose microRNA signatures—alongside thorough phenotyping of diet, gut hormones, and body composition—are optimally positioned for future advancements.

Although our analysis focusses on germline changes in the leptin melanocortin pathway, postoperative results may also be affected by miRNA-mediated regulation. Nutrition and exercise affect several miRNAs, which in turn influence tissue remodelling, inflammation, and cellular metabolism. Stimulus-responsive miRNAs, including miR-21, miR-221-3p, and miR-222-3p, illustrate the capacity of environmental factors to modify transcriptional programmes linked to weight loss and glycaemic improvement, as they are involved in pathways governing differentiation, insulin signalling, and energy metabolism. Recent studies demonstrate that “exercise and nutrition regulate miRNA expression with functional consequences for metabolic tissues”, hence reinforcing the notion of gene-environment interaction [88]. This indicates that lifestyle-induced miRNA modifications may amplify or diminish allelic effects at the *LEP/LEPR*, *POMC*, and *MC4R loci.* This paradigm suggests that structured food and exercise treatments may influence epigenetic mechanisms on key neuro-endocrine pathways after surgery, explaining the differing outcomes of individuals with similar genotypes. These interactions may be measured in prospective studies that combine genotyping with objective evaluations of diet and exercise, as well as sequential miRNA profiling in blood and adipose tissue (both pre-operative and 6–12 months post-operative).

We integrated recent syntheses with fundamental mechanistic references to contextualise route biology within modern human genetics. A 2024 comprehensive review consolidates variant–outcome correlations after metabolic or bariatric surgery, emphasising regions of consistent findings and those with initial evidence. Overviews of precision medicine discuss the potential integration of genetic data with clinical predictions; however, they also indicate that impact sizes are minimal and context-dependent. Ultimately, concentrated investigations of leptin–melanocortin mutations reveal minor, persistent alterations in postoperative weight patterns. This substantiates biological plausibility but does not advocate for therapeutic use. These modifications together maintain an appropriate level of caution while redirecting our attention to the literature from 2023 to 2025 [75,80].

Our research focusses on germline changes in the leptin melanocortin pathway, while postoperative results may also be affected by miRNA-mediated control. Stress responsive miRNAs, including miR-21, miR-221-3p, and miR-222-3p, illustrate the alteration of transcriptional programmes linked to energy balance and insulin signalling as a result of lifestyle variables. Diet and exercise alter miRNA expression in real time, impacting metabolism and tissue remodelling. These findings illustrate a widely relevant epigenetic process via which lifestyle variables affect gene expression networks, whereas skeletal and stromal models provide substantial direct evidence. This mechanism may engage with allelic variation at *LEP/LEPR*, *POMC*, or *MC4R* to enhance or diminish weight-loss and glycaemic trajectories after surgery. To quantify these interactions and improve risk classification, prospective designs that include genotyping with objective dietary and activity evaluations, as well as serial miRNA profiling in blood and adipose tissue (pre-operative and 6–12 months post-operative), are ideal.

To ensure clarity and scientific integrity, we deliberately preserved a more extensive mechanistic perspective. A pathway-level map of the leptin melanocortin axis (*LEP/LEPR*, *POMC*, *MC4R*, and *FTO*) improves clarity and minimises the potential for misinterpretation, enabling non-specialist readers, such as physicians, dietitians, and surgeons, to associate diverse study results with particular biological nodes and causal pathways. The anticipated clinical outcome (diminished weight loss or gradual glycaemic enhancement) is interpretable a priori rather than post hoc when variations converge on diminished melanocortin signalling. This is the second manner in which mechanistic parsimony elucidates facts that seem contradictory. Third, bariatric surgery induces coordinated changes in inflammation, bile acids, gut hormones, and energy regulation. In the absence of a comprehensive mechanistic framework, the integration of data across research may regress to simple correlations without a valid causal connection. This framework ultimately improves repeatability and supports translational goals by connecting genotype (or epigenotype) to therapeutic treatments, including procedure selection, dietary counsel, and physical exercise.

We amalgamate this mechanistic framework with cross-study synthesis in the Results/Discussion (study counts, total N, effect directions, and sources of heterogeneity) to attain a balance between synthesis and profundity. This approach of mechanism-guided evidence analysis maintains clinical relevance, enhances transparency, and mitigates over-interpretation that may arise from data aggregation based only on narratives.

Our synthesis does not advocate for the clinical use of genotypes in the selection of applicants or procedures. If verified, any further use of genetic data would enhance, rather than replace, recognised factors influencing outcomes, such as caloric intake and expenditure, compliance with dietary and physical activity recommendations, surgery anatomy, and postoperative care. Genetics should continue as a field of investigation and hypothesis development until strong prospective validation is attained.

### 4.5. Limitations and Implications

Limited sample sizes, heterogeneity among studies (including divergent definitions of outcomes such as TBWL, %EWL, and glycaemic composites; inconsistent follow-up durations; and diverse surgical techniques), along with non-standardised genotyping and analytical methodologies (encompassing variant coverage, genotype coding models, various platforms, and inconsistent adjustment for confounders) constrain inference. Eliminating possible sample overlap is not always practicable, and variance-qualified figures are not uniformly given. To prevent misleading accuracy, these elements facilitate a direction-of-effect synthesis and negate a steady pooled effect. Ultimately, inferences related to fertility are tentative owing to the sparse recording of genotype-stratified reproductive outcomes.

A pre-registered, standardised framework that (i) delineates uniform procedural strata, follow-up intervals (e.g., 6, 12, 24 months), and primary outcomes (preferably total body weight loss alongside a minimal glycaemic set), (ii) enforces consistent genotyping and coding with explicit adjustments, and (iii) prospectively collects genotype-stratified reproductive endpoints (ovulation, menstrual regularity, anti-Müllerian hormone, sex-steroid profiles, and time-to-pregnancy/IVF indices) is proposed. Such designs may elucidate which pathway-anchored hypotheses transition from biological plausibility to empirical support.

We intentionally refrain from providing clinical recommendations to prevent overreach, and we emphasise that, due to sample sizes, heterogeneity, and non-standardised methodologies, genotype-guided management is premature.

## 5. Conclusions

The varied outcomes of weight loss following bariatric surgery illustrate the complexity and multifactorial nature of obesity, which is significantly influenced by genetic factors affecting the neural and peripheral systems that regulate energy consumption, expenditure, and storage. This review demonstrates how genetic variations, particularly in FTO, MC4R, LEPR, and POMC, interact with surgical techniques to influence both short-term and long-term metabolic alterations post-surgery. Mutations or polymorphisms at these loci not only indicate an individual’s susceptibility to obesity but also disrupt thermogenesis, hypothalamic satiety signalling, adipose tissue regulation, and gut–brain interactions.

An increasing body of evidence indicates the necessity to transcend universal surgical paradigms. Patients possessing loss-of-function mutations in the *LEP–LEPR–POMC–MC4R* pathway may exhibit diminished responsiveness and duration to restricted or malabsorptive interventions due to their hypothalamus’s inability to adequately process peripheral satiety signals. FTO polymorphisms appear to influence thermogenic plasticity and mitochondrial metabolism, complicating the body’s ability to sustain energy expenditure post-surgery. The pathophysiological constraints underscore the necessity of categorising surgical candidates by genotype and modifying therapies accordingly.

The integration of genetic screening, polygenic risk scores, and multi-omic data, including epigenetic, transcriptomic, and metabolomic profiling, enables unprecedented precision in bariatric surgery. The integration of machine learning and artificial intelligence with extensive genomic databases will enable the development of predictive algorithms capable of forecasting individual outcomes, identifying those prone to weight regain, and assisting with adjunctive medication. 

From a translational perspective, the future of bariatric care encompasses a model that integrates genetic counselling and culminates in a tailored surgical and pharmacological treatment strategy. The implementation of genotype-based surgical decision-making, central and peripheral biomarkers, and AI-driven algorithms for dynamic monitoring will redefine success in the treatment of obesity. Ultimately, metabolic surgery will be effective in the long term only if the molecular deficiencies responsible for energy dysregulation are rectified alongside the physical alterations.

At present, there is little information to guide therapeutic decision-making; yet, heterogeneity in the leptin–melanocortin axis may modestly influence surgical weight-loss trajectories in context-dependent ways. According to the available facts, we do not endorse regular preoperative genotyping or the selection of operations based on genotype. The results of bariatric surgery, an exceptionally efficient weight control approach, eventually take into account energy balance as well as behavioural, anatomical, and physiological aspects. Genetics should now be seen as modifiers that generate ideas rather than as determinants for practical interventions. Before genotype-informed risk classification can be judiciously considered, further research that are well-coordinated and sufficiently powered are essential.

## Figures and Tables

**Table 1:** Systematic Search Strategy Across Databases (PubMed, Scopus, and Web of Science).

Database	Search String	Date of Search	Filters Applied
PubMed	(“Roux-en-Y gastric bypass” OR “RYGB” OR “bariatric surgery”) AND (“genetic variant” OR “polymorphism” OR “SNP” OR “FTO” OR “MC4R” OR “LEPR” OR “SH2B1” OR “POMC” OR “PCSK1” OR “SIM1”) AND (“weight loss” OR “BMI” OR “%EWL” OR “%TBWL” OR “weight regain”)	April 2025	Humans, English, Full-text, All years
Scopus	TITLE-ABS-KEY((“Roux-en-Y gastric bypass” OR “RYGB” OR “bariatric surgery”) AND (“genetic variant” OR “SNP” OR “FTO” OR “MC4R” OR “LEPR”) AND (“weight loss” OR “BMI” OR “%EWL” OR “%TBWL” OR “weight regain”))	April 2025	English, Peer-reviewed, All years
Web of Science	TS = (“Roux-en-Y gastric bypass” OR “RYGB” OR “bariatric surgery”) AND TS = (“genetic variant” OR “SNP” OR “FTO” OR “MC4R” OR “LEPR”) AND TS = (“weight loss” OR “BMI” OR “%EWL” OR “%TBWL” OR “weight regain”)	April 2025	English, Original articles, All years

**Table 2:** Detailed Risk of Bias Assessment of the Included Studies Using the Newcastle–Ottawa Scale (NOS).

Study	SEL1: Representativeness	SEL2: Non-Exposed Selection	SEL3: Exposure Ascertainment	SEL4: Outcome Not Present at Start	COMP1: Confounder Adjustment	COMP2: Additional Adjustment	OUT1: Outcome Assessment	OUT2: Follow-Up Duration	OUT3: Adequacy of Follow-Up	Total NOS Score	Risk of Bias
De Luis et al., 2012, [42]	1	1	1	1	1	0	1	1	1	8	Low
Balasar et al., 2015, [43]	1	1	1	1	1	0	1	1	1	8	Low
Bandstein et al., 2015, [13]	1	1	1	1	1	0	1	1	1	8	Low
Perez-Luque et al., 2024, [44]	1	1	1	1	1	0	1	1	1	8	Low
Rodrigues et al., 2015, [45]	1	1	1	1	1	0	1	1	1	8	Low
Campos et al., 2022, [17]	1	1	1	1	1	0	1	1	1	8	Low
De Luis et al., 2010, [46]	1	1	1	1	1	0	1	1	1	8	Low
Kops et al., 2018, [47]	1	1	1	1	1	0	1	1	1	8	Low
Li et al., 2019, [48]	1	1	1	1	1	0	1	1	1	8	Low
Cooiman et al., 2020, [49]	1	1	1	1	1	0	1	1	1	8	Low
Valette et al., 2012, [5]	1	1	1	1	1	0	1	1	1	8	Low
Aslan et al., 2011, [7]	1	1	1	1	1	0	1	1	1	8	Low
Mirshahi et al., 2011, [50]	1	1	1	1	1	0	1	1	1	8	Low
Moore et al., 2014, [20]	1	1	1	1	1	0	1	1	0	7	Low
Potoczna et al., 2004, [51]	1	1	1	1	1	0	1	1	1	8	Low
Salazar-Valencia et al., 2022, [52]	1	1	1	1	1	0	1	1	1	8	Low
Novais et al., 2021, [53]	1	1	1	1	1	0	1	1	1	8	Low
Resende et al., 2017, [54]	1	1	1	1	1	0	1	1	1	8	Low
Novais et al., 2022, [53]	1	1	1	1	1	0	1	1	0	7	Low
Gong et al., 2023, [55]	1	1	1	1	1	0	1	1	1	8	Low

We employed the Newcastle–Ottawa Scale to evaluate the quality of each study. This scale examines three domains: Selection (4 items), Comparability (2 things), and Outcome (3 items). Every study received a rating ranging from 0 to 9. Studies scoring 7 or above were considered to have a low risk of bias, those scoring 5 or 6 were deemed to have a moderate risk, and those scoring below 5 were classified as having a high risk.

**Table 3:** Methodological Characteristics of the Included Studies Investigating Genetic Variants and Bariatric Surgery Outcomes.

Year	Author	Type of Study	Inclusion Criteria	Exclusion Criteria	Female Sample Size	Gene Investigated	Polymorphism Investigated	Genotypes Investigated	Follow-Up	Bariatric Surgery Performed	Results
2012	De Luis et al. [42]	Case–control study	BMI > 40 BPD operation performed	Loss to follow-up	87	FTO	rs9939609	TT: wild type AT/AA: mutant type	3, 9 and 12 months	BPD	Higher initial weight loss at 3 months in carriers of TT variant (wild type) of FTO gene At 9 and 12 months after BPD the weight loss was similar in both genotypes(TT, AT, AA)
2015	Balasar et al. [43]	Case–control study	BMI ≥ 40 LSG operation performed	Loss to follow-up	54	FTO	rs9939609	TT: wild type AT/AA: mutant type	6 months	LSG	Percent of excess weight loss at 6 months of follow-up was similar in both wild type (TT) and mutant (AT, AA) groups rs9939609 FTO gene polymorphism is not a useful genetic test in predicting the weight loss
2015	Bandstein et al. [13]	Case–control study	BMI ≥ 40 RYGB performed	Loss to follow-up	151	FTO	rs9939609	TT: wild type AT/AA:mutant type	2 years	RYGB	Patients who were vitamin D-deficient prior to surgery exhibited a ~14% higher RYGB surgery induced weight loss when they carried two copies of the A-allele compared to vitamin D-deficient patients who were homozygous for the FTO T-allele
2024	Perez-Luque et al. [44]	Retrospective analysis	BMI ≥ 40 RYGB performed	Loss to follow-up Informed consent not obtained	78	MC4R FTO	MC4R: rs17782313 FTO: rs9939609, rs9930506, and rs1421085	rs9939609: TT, TA and AA rs9930506: AA, AG and GG rs1421085: TT, TC and CC	4 to 8 years	RYGB	No association was found between the MC4R polymorphism and total body weight loss, post-surgery weight, and BMI after bariatric surgery.
2015	Rodrigues et al. [45]	Case–control study	BMI ≥ 40 RYGB performed	Loss to follow-up Inconclusive genotyping Informed consent not obtained	124	FTO	rs9939609	TT: wild type AT/AA: mutant type	5 years	RYGB	FTO AA or AT genotypes does not influence weight until 2 years after surgery Weight loss was lower in FTO group starting 2 years after surgery Weight regain was higher and earlier in FTO group
2022	Campos et al. [17]	Case–control study	>18 years old RYGB performed Genomic DNA available for analysis Patients carriers of a heterozygous variant in the leptin-melanocortin pathway	Less than 6 months from RYBG A bariatric reintervention or revisional procedure Use of an antiobesity medication after RYBG Pregnancy after RYBG	118	MC4R FTO POMC LEPR	MC4R: p.Leu325Phe, p.Gln156 LEPR: p.Cys954Phe, p.Arg612His, p.Val144Leu, p.Tyr747Asp, p.Ser950Thr, p.Arg514Gly, p.Val344Ile, p.Val984Ala, p.Pro401Leu. POMC: p.Pro194Ala, p.Phe144Leu, p.Cys5Tyr, p.His143Gln, p.Arg48Gln, p.Arg236Gly, p.Thr39Met, p.Pro132Ala, p.Tyr221Cys	N/A	1, 3, 6, 12, and 18 months and then yearly from year 2 to 15	RYGB	Patients carriers of a heterozygous variant in the leptin-melanocortin pathway have a lower weight loss and a higher weight regain after RYGB
2010	De Luis et al. [46]	Case–control study	BMI > 40 BPD operation performed	Loss to follow-up	32	LEPR	rs1805094	Lys656Asn and Asn656Asn: mutant group Lys656Lys: wild-type group	3, 9, 12 months	BPD	Weight loss was higher in mutant group than wild-type group after bariatric surgery
2018	Kops et al. [47]	Prospective cohort study	BMI > 40 RYGB operation performed	<18 years old Presence of current suicide risk Hospitalization for psychiatric reasons or intellectual disability or dementia. Pregnant or lactating women during the 5 years postop period	92	FTO LEPR	FTO: rs9939609 LEPR: rs1137101	FTO: AA, TA, TT LEPR: AA, AG, GG	3, 6, 12, 24 months	RYGB	No effect on weight loss or clinical outcomes after bariatric surgery in FTO gene polymorphism varriers. The AA genotype of the rs1137101 polymorhism seems to be associated with a higher weight loss
2018	Li et al. [48]	Case–control study	15–55 years old BMI > 28 LSG operation performed	Loss to follow-up Informed consent not obtained	6	POMC LEP/LEPR MC4R	LEP/LEPR: H118L, A1033T, Q463X MC4R: C277X, V166I, c.1350delA, V166I	N/A	3, 6, 12, 18, and 24 months and then yearly until year 5	LSG	At 18 months, mutation carriers demonstrated a lower maximum weight loss value Mutation carriers demonstrated difficulties in maintaining their weight loss
2019	Cooiman et al. [49]	Case–control study	18–65 years old Indication for bariatric surgery (BMI > 50, childhood onset obesity and/or indication for revisional surgery)	Loss to follow-up Informed consent not obtained	Not reported	POMC MC4R	Not reported	N/A	12 and 24 months	Primary Gastric Bypass or RYGB	Carriers of mutations in MC4R and POMC did not demonstrate different total body weight loss after RYGB compared to non-carriers Carriers of MC4R mutations showed significantly lower total body weight loss after sleeve gastrectomy compared to non-carriers During the 2-year follow-up period
2012	Valette et al. [5]	Case-control study	BMI > 28 RYGB or AGB was performed	Informed consent not obtained	123	MC4R	V103L I251L rs17782313	N/A	3, 6 and 12 months	RYGB or AGB	Weight loss at 3, 6 and 12 months did not differ between carriers and non-carriers irrespective of the MC4R mutation
2011	Aslan et al. [7]	Case–control study	BMI > 40 BMI > 35 and <40 accompanied by obesity-related comorbidities RYGB was performed	Informed consent not obtained	7	MC4R	Cys271Phe Gln307stop Arg236Cys	N/A	1,3, 6, 9, and 12 months	RYGB	Weight loss after RYBG is independent of the presence of MC4R mutations
2011	Mirshahi et al. [50]	Case–control study	BMI > 40 RYGB was performed	Pregnancy after surgery	1146	MC4R	rs52820871 rs2229616	N/A	Up to 48 months	RYGB	Carriers of I251L mutation demonstrated better weight loss after surgical interventions
2014	Moore et al. [20]	Case–control study	BMI > 40 RYGB was performed	Informed consent not obtained	1146	MC4R	V95I I137T L250Q	N/A	Up to 84 months	RYGB	Carriers of V95I, I137T and L250Q mutation demonstrated worse weight loss after surgical interventions
2004	Potoczna et al. [51]	Case–control study	BMI > 35 >18 years old, <70 years old Laparoscopic gastric banding was performed	Informed consent not obtained Alcohol or substance abuse	233	POMC LEP/LEPR MC4R	POMC: C4512T Cys6Cys 0.007, C7662T, C7965T, C4335G, A7429G, C7726T, C7774G, A8021G, A8042G, C8086G, C8246T LEPR: T88641C, G88642A, G88917A, T88928C, C95778T, T95869C, T96008C, T96135C, A96215G, A97118G, G97244A MC4R: rs199862517, rs13447329, rs13447332, rs2229616, rs52820871, NM_005912.3c.544T>C, NM_005912.3c.991A>G, NM_005912.3c.1419A>G	N/A	36 months	Laparoscopic gastric banding	Carriers of MC4R mutations demonstrated less weight loss compared to non-carriers
2022	Salazar-Valencia et al. [52]	Case–control study	BMI > 35 >18 years old RYGB was performed	Informed consent not obtained	206	MC4R	Ile269Asn	N/A	6 months	RYGB	Carriers of the MC4R polymorphism demonstrated similar weight loss with the non-carriers
2021	Novais et al. [53]	Case–control study	18–50 years old BMI ≥ 40 or ≥35 kg with associated comorbidities RYGB performed	Alcohol or substance abuse Genetic syndromes associated with obesity Cushing’s syndrome Hypothyroidism renal or liver failure neoplasia HIV infection Postmenopausal oestrogen replacement therapy Corticosteroids use	95	LEP/LEPR FTO	LEP/LEPR: rs7799039, rs1137101 FTO: rs9939609	N/A	12 months	RYGB	Carriers of mutated variants did not demonstrate statistically significant differences in energy intake 1 year postop compared to non-carriers
2017	Resende et al. [54]	Retrospective cohort	>18 years old RYGB performed	Informed consent not obtained Metabolic or inflammatory diseases or dietary habits alterations postop	141	MC4R	rs17782313	CT + CC: carriers TT: non-carriers	6, 9, 12, 18, 24, 36, 48 and 60 months	RYGB	Carriers of rs17782313 MC4R polymorphism demonstrated an increased risk not to reach BMI < 30, tending to maintain a BMI > 35
2022	Novais et al. [56]	Case–control study	18–50 years old BMI ≥ 40 or ≥35 kg with associated comorbidities RYGB performed	Alcohol or substance abuse Genetic syndromes associated with obesity Cushing’s syndrome Hypothyroidism renal or liver failure neoplasia HIV infection Postmenopausal oestrogen replacement therapy Corticosteroids use	351	LEP/LEPR FTO	LEP/LEPR: rs7799039, rs1137101 FTO: rs9939609	N/A		RYGB	Carriers of mutated variants did not demonstrate excessive weight loss 1 year compared to non-carriers
2023	Gong et al. [55]	Retrospective cohort	>18 years old BMI ≥ 45 Bariatric surgery performed	Informed consent not obtained	Not reported	MC4R	Y35C T53I V103I R165W G233S C277X	N/A	1, 3, 6, 12, 18, and 24 months and yearly thereafter	RYGB or sleeve gastrectomy	Carriers of R165W and G233S variants demonstrated increased excess weight loss postsurgery

This table provides a comprehensive overview of 20 studies examining the association between gene polymorphisms and weight loss following bariatric surgery. The document encompasses the publication year, the author, the study design, the inclusion and exclusion criteria for participants, the number of female participants, the genes and specific polymorphisms examined, the genotypes, the duration of follow-up, the type of surgery performed, and the primary findings regarding the differential weight loss patterns between carriers and non-carriers. We examined genetic targets within the leptin–melanocortin axis and the mechanisms regulating adipose tissue accumulation. The outcomes were varied regarding the accuracy of predictions for weight changes post-surgery.

## Data Availability

All data supporting the findings of this review are available within the cited published studies. No new datasets were generated or analysed during the current work.
